# Influence of Mixed Imide Composition and Thermal Annealing on Ionic Liquid Uptake and Conductivity of Polyimide-Poly(ethylene glycol) Segmented Block Copolymer Membranes

**DOI:** 10.3390/molecules26247450

**Published:** 2021-12-09

**Authors:** Gokcen A. Ciftcioglu, Curtis W. Frank

**Affiliations:** 1 Department of Chemical Engineering, Marmara University, Istanbul 34722, Turkey; 2 Department of Chemical Engineering, Stanford University, Stanford, CA 94305, USA; cwfrank@stanford.edu

**Keywords:** copolymerization, bicontinuous structures, ionic liquid, polymer exchange membranes

## Abstract

Understanding the impact of different bridging groups in the two-step polymerization of poly(ethylene glycol) (PEG)-incorporated polyimide (PI) materials is significant. It is known that the proton exchange membranes (PEMs) used in industry today can experience performance degradation under rising temperature conditions. Many efforts have been devoted to overcoming this problem by improving the physical and mechanical properties that extend the hygrothermal life of a PEM. This work examines the effect of oxygenated and fluorinated bridging anhydrides in the production of PI-PEG PEMs. It is shown that the dianhydride identity and the amount incorporated in the synthesis influences the properties of the segmented block copolymer (SBC) membranes, such as increased ionic liquid uptake (ILU), enhanced conductivity and higher Young’s modulus favoring stiffness comparable to Nafion 115, an industrial standard. Investigations on the ionic conductivity of PI-PEG membranes were carried out to determine how thermal annealing would affect the material’s performance as an ion-exchange membrane. By applying a thermal annealing process at 60 °C for one hour, the conductivities of synthesized segmented block copolymer membranes values were increased. The effect of thermal annealing on the mechanical properties was also shown for the undoped SBC via measuring the change in the Young’s modulus. These higher ILU abilities and mechanical behavior changes are thought to arise from the interaction between PEG molecules and ethylammonium nitrate (EAN) ionic liquid (IL). In addition, higher interconnected routes provide a better ion-transfer environment within the membrane. It was found that the conductivity was increased by a factor of ten for undoped and a factor of two to seven for IL-doped membranes after thermal annealing.

## 1. Introduction

The continuously growing need for additional energy sources along with sustainable management at the same time has induced higher demand for renewable energy technologies. However, a variety of renewable energy applications, such as solar, wind, biofuel, nuclear, geothermal, and hydrogen, have financial, technical, and environmental challenges, making their management even harder [1,2]. Depending on their energy source, some of the technologies, such as solar and wind, will not be able to continuously supply reliable energy, especially during the nighttime when peak energy demand increases. Thus, the storage of energy to be used on-demand, when an energy source is not available, creates the need to use multiple energy technologies [3,4]. Because of both technical and financial difficulties in integrating multiple energy technologies, there is a trend toward systems where the energy can be produced and stored at the same time [4,5]. Fuel cells with these abilities have generated considerable interest. Proton exchange membrane fuel cells (PEMFCs) have been tracked by the industry, and considerable knowledge has been accumulated on the membranes, catalysts, fuels, and oxidants that are well-established [6,7,8].

Membranes being used as the separator in PEMFCs must satisfy unique characteristics simultaneously, such as having high proton conductivity but low electronic conductivity, low fuel and oxidant permeability, limited crossover and solvent transport, thermal and oxidative stability, low cost and good mechanical properties, and easy fabrication of fuel cells [1,8]. The state-of-the-art perfluorosulfonic acid (PFSA) membranes, such as Nafion^®^, have successfully been operated in fuel cell applications [9]. However, the temperature range in these systems is limited to 50–90 °C due to the need to keep Nafion^®^ hydrated [5,8,9]. Low-temperature operation conditions increased the concerns, especially with regard to the cost [7]. Thus, continuous efforts are still being made in the development of proton exchange membrane (PEM) technology for operation above 120 °C [10,11,12,13,14]. However, increasing the temperature causes the stability of the membrane to decrease, and sustaining hydration and ionic conductivity become other challenges to overcome [8].

Composites and membranes that have been investigated include other PFSA membranes, styrene-based systems, polyphosphazenes, polybenzimidazoles, sulfonated poly ether ether ketones (SPEEK), sulfonated polyimides (PI), poly(ethylene glycol) (PEG), and polyimides and polyimide composites with various additives, such as silica, carbon nanotubes, etc. [12,13,14,15,16,17,18,19,20,21,22,23,24,25,26,27,28,29]. All these polymer systems (and Nafion^®^ membranes and their derivatives as well) have been evaluated to achieve a deeper understanding of transport properties, confinement properties, proton conductivities, and thermal stabilities [29,30,31,32,33,34,35,36,37,38,39,40,41,42,43,44,45]. Especially for the alternative membranes and composites, one of the most urgent challenges remaining to be overcome, and the most significant barrier to their successful operation, is the development of a polymer electrolyte that maintains the proton conductivity and proper mechanical strength at elevated temperatures. 

Recent studies by Coletta et al. showed that polyimide polyethylene glycol (PI-PEG) segmented block copolymer (SBC) systems have the potential to be used as proton exchange membranes [13,28,34]. In these SBC systems, aromatic PIs were used along with PEGs of varying molecular mass to produce PI-PEG SBCs with phase-separated PEG domains. These domains play crucial roles in affecting conductivity. When swollen with ionic liquid (IL), these domains develop interconnected micro- or nano- channels, creating easier pathways for ion transfer. In order to study the possible formation of these channels, in other words, the effect of IL on the structure, small-angle X-ray scattering (SAXS) measurements were taken for several varieties, such as different PEG concentrations (0–50 wt%) [28], different PEG molecular weights (990–6000 g/mol) [28], changing the solvent (*N*,*N*-dimethylacetamide and 1-methyl-2-pyrrolidinone) as the synthesis environment [34], and changing either the fluorinated or oxygenated base diamines or dianhydrides [13,34].

These advanced studies showed that polymer–polymer interactions and changes to the structure at the molecular level altered the rigidity, flexibility, and length scale of PEG domains or aromatic regularity of the resulting membranes, where the impacts of these changes on the conductivity were presented [13,28,34]. A maximum of 102 milliSiemens/centimeter (mS/cm), an increase of 28% in conductivity compared to using fluorinated diamine while using fluorinated dianhydride, was observed for the following conditions for an ethylammonium nitrate (EAN)-doped membrane sample: 80 °C and 70% relative humidity (RH), using fluorinated dianhydride, 50 wt% PEG (Mn~1500) (PEG 1500) and oxygenated diamine. It was shown via SAXS results that this positive impact on the conductivity was due to the formation of more well-defined doped polymer ionic pathways through the material by the more planar, less bulky oxygenated diamine [13]. 

The conductivity change for EAN-doped samples when switching from oxygenated dianhydride to the fluorinated dianhydride SBC system synthesized using 50 wt% PEG (Mn~1500) was, on average, 15%. This lower increase in conductivity when switching dianhydrides was shown via SAXS experiments to be due to the bulky nature of the fluorinated dianhydride, which interferes with the definition of ionic pathway boundaries [13]. 

Coletta et al. further showed that an increase in the molecular weight of PEG in PI-PEG SBC systems produced more disordered structures with increasing randomly distributed PEG domains [28]. More ill-defined PEG domains for the highest molecular weight of PEG (Mn~6000) incorporated into the system were formed, as shown in SAXS figures by Coletta et al., with higher correlation length which can be seen as evidence of undesirable PEG crystallinity in PEMs and large scale phase-separation of PEG [28].

The work of Woo et al. focused on PI-PEG SBC systems synthesized from fluorinated dianhydride, oxygenated diamine, and PEG1500 [39]. Systems with varying amounts of PEG1500 weight percentages of 0%, 11.3%, 26.2%, 33.6%, 42.1%, and 46.8% were studied to augment the work of Coletta et al., where only weight ratios of 30 %, 40%, and 50% were investigated for PEG1500 [28]. Woo et al. studied the structural change of these six PI-PEG SBC systems via SAXS for both undoped and EAN-doped samples [39]. In their study, it was shown that increasing the PEG 1500 content beyond 33% produced a more ordered structure and more defined PEG+EAN domains. This enhanced order was associated with higher conductivity, e.g., of 209 mScm^−1^ for 42.1 wt% PEG 1500. 

The results obtained by Woo et al. are comparable with that of Nafion^®^, so we infer that the PEG1500-incorporated PI family has potential for use as PEMS. However, further evaluation of the mechanical properties of the membrane system comprising the above-mentioned aromatic dianhydrides, diamines, and PEG1500 is needed. Synthesis of more stable, flexible, homogenous membranes with high conductivities that are compatible with commercially available Nafion^®^ and are stiff enough to be handled is the main goal of this study.

The present work examines the specific polyimides synthesized with a mixture of two various dianhydrides (ODPA and 6FDA) with emphasis on their temperature-dependent morphology and conductivity. The effects of ionic liquid uptake (ILU) and water uptake (WU) on these properties are studied at a temperature of 25 °C. Our studies clearly show that 1 h of thermal annealing after the synthesis of the membranes (undoped and doped in ILU) prior to conductivity measurements resulted in a significant increase in their conductivities with the doped case higher than the undoped case. It is known that aromatic interactions play a crucial role in the formation of PEG-EAN domains with well-defined boundaries [13]. Thus, we suggest that the increased uptake of EAN at a higher temperature coupled with more well-defined PEG-EAN domains and the resulting increase in the number of possible ion-transfer interconnected routes are responsible for the observed increase in the conductivity. 

## 2. System Design

This research was motivated by the need for a better material to be used at elevated temperatures above 100 °C which can maintain good mechanical properties and yet provide an environment for effective and high electron transfer via possessing less resistivity. As reported in Coletta et al. and Woo et al.’s studies [13,28,39], there are still areas to be fixed in SBC-type membranes in order to be used in industry. Therefore, the objective of this study was to find an optimal material composition for the PI-PEG SBC membrane class.

As mentioned earlier, studies by Coletta et al. showed that PEG-PI systems have the potential to be used as proton exchange membranes (PEMs). Extensive research on this type of material has been conducted, and structural and morphological analyses are given for SBC membranes consisting of either oxygenated or fluorinated diamines along with oxygenated or fluorinated dianhydrides. Through the research performed by this group, it has been shown that the morphology and the free volume domains can be affected depending on the composition. Thus, Coletta et al. concluded that by changing the type of the PI system in PEG-PI SBC membranes, more desirable and superior materials can be produced.

Further investigations were carried out by Woo et al. [39], where it was shown that the morphology of the PI-PEG system shows the same pattern as disordered bicontinuous phase-separated nanostructures, which were described by the Teubner–Strey theory. These authors reported that these bicontinuous phase-separated nanostructures positively affected the conductivity and showed increased ionic conductivity by two to five times compared to that of Nafion films. Moreover, the temperature annealing process was performed for three different temperatures of 100, 120, and 140 °C to study the temprature’s effect on the nanostructure and conductivity. Woo et al. [39] showed that the temperature annealing process increased conductivity by about four times compared to that of Nafion for SBC membranes with PEG compositions above 26 wt%.

After careful analysis of all of these extensive studies, we were motivated to acquire more information by varying the composition of PI-PEG SBC membranes. Thus, in this study, two groups of PI-PEG SBC membranes were synthesized.

In the first group, PI-PEG SBC membranes containing both fluorinated and oxygenated dianhydrides were produced using different compositions. The systematic experimental design is shown in Table 1, along with the acronyms of the synthesized membranes. 

Additionally, to gain more insight into the effect of changing imide composition in the final product, different dianhydride concentrations were examined, keeping PEG1500 and PDODA contents constant at 42.1 and 27.9 wt%, respectively. Table 2 summarizes the second group of SBC membranes synthesized for this investigation.

## 3. Results

### 3.1. Synthesis of Polyimide

To verify the production of the polyimides via the two-step polymerization process, FTIR measurements were first performed on the PAA solutions and the resulting membranes after thermal processing. 

First, analyses were conducted for the series of polyimide systems with varying PEG contents in the membrane. Supplementary Figures S1–S3 show FTIR spectra of the polyimide-poly(ethylene glycol) membranes listed in Table 1 and the corrected height of the peaks to observe the effects of changing PEG concentration (28.8 wt%, 42.1 wt%, and 46.8 wt%) in the system. In addition, FTIR analyses were conducted for the second set of membrane families listed in Table 2 containing varying amounts of the dianhydrides and a fixed PEG content at 42.10 wt%. To represent the influence of the dianhydrides, the FTIR spectra for these systems before and after imidization are shown in Supplementary Figures S4–S8. 

The success of the synthesis can be seen from the peaks around 1715 cm^−1^, which result from the > C=O groups of the polyimide, and peaks around 1500 and 1409 cm^−1^, which represent the stretching of carbonyl groups in the polyimide [13,42,43,44]. Supplementary Figures S4–S8 show the reduction in the intensity of the peaks around 2930, 1635, and 1548 cm^−1^, which suggests that the imidization process took place in the two-step polymerization [34,44,45].

Supplementary Figures S1–S3 show the effects of increasing the proportion of PEG concentration. As the PEG content increases in the copolymer system, by analyzing the height of the peaks, it may be concluded that increasing PEG concentration enhances the yield of the polyimides with more ordered PEG domains. This may be the reason for the peaks seen in SAXS experiments studied in the literature [34]. However, this feature of the reaction has to be investigated more thoroughly and will not be discussed further. 

### 3.2. Material Characterization

#### 3.2.1. Thermal Stability of PI-PEG Systems

Membranes must withstand the elevated temperatures around 120 °C of operating fuel cells. To evaluate our PEMs’ thermal stability, thermal gravimetric analysis (TGA) was performed. Thus, the aim of this experiment was to present the thermal stability of the synthesized membranes for operation temperatures around 120 °C.

**Figure 1:**
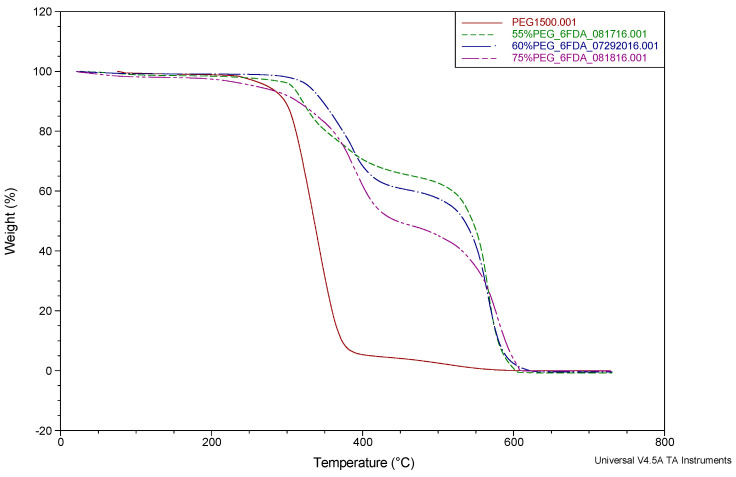
TGA of 6FDA-PDODA-PEG1500 (changing PEG content) SBC membranes (percentages given in the figure for PEG content are mol%).

**Figure 2:**
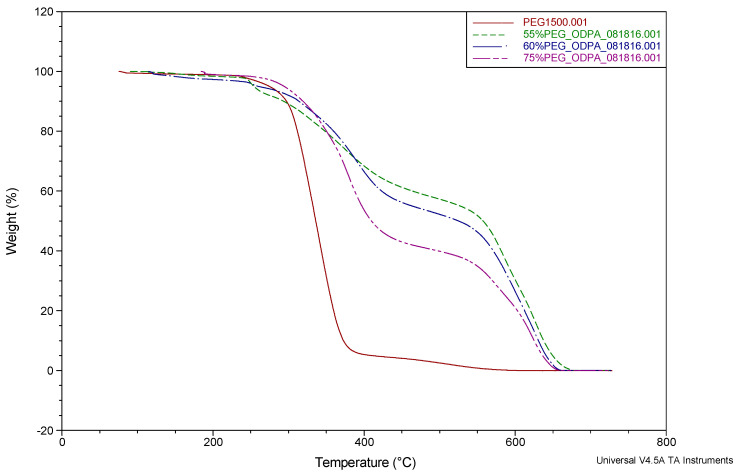
TGA of ODPA-PDODA-PEG1500 (changing PEG content) SBC membranes (percentages given in the figure for PEG content are mol%).

**Figure 3:**
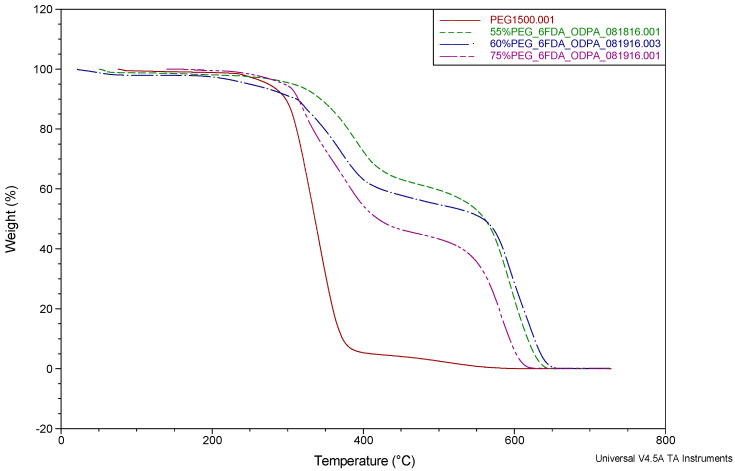
TGA of 6FDA + ODPA-PDODA-PEG1500 (changing PEG content) SBC membranes (percentages given in the figure for PEG content are mol%).

The TGA results of all the synthesized membrane families are shown in Figure 1, Figure 2 and Figure 3. 

Previous works reported by Coletta et al. [13,28,34] and other studies in the literature [46,47,48,49] showed that their PEG-incorporated polyimide copolymer systems realized a mass loss around 250–450 °C. The new SBC membranes described here showed a similar mass loss in the range of 300–550 °C, occurring in two stages. The first major feature starting around 300 °C is due to the loss of PEG1500 content. The weight loss for PEG1500 content was found to be consistent with the incorporated amounts of 28.8, 42.1, and 46.8 wt%. To understand the weight-loss behaviors, the TGA of PEG1500 is also presented in Figure 1, Figure 2 and Figure 3 (red line). It can be seen that PEG1500 starts to degrade at 300 °C and completely degrades at around 400 °C. In the new membranes, another mass loss can be seen around 550 °C, which is the degradation temperature for the remaining aromatic polyimide content of the SBC membrane. These observed results for the degradation of PI-PEG SBC systems are in line with the literature [34,46,47,48,49]. It can be concluded that these membranes are stable enough for practical applications with operating conditions above 100 °C.

#### 3.2.2. Morphology of Polymers

To understand how conductivity and flexibility are affected by the membrane composition, thermal measurements can be an important guide. Thus, differential scanning calorimetry (DSC) analysis was conducted. 

The PI-PEG segmented membrane copolymer systems did not show any of the endotherms related to PEG crystallinity, which are in line with the previous studies [13,28,34,39]. 

#### 3.2.3. Ionic Liquid and Water Uptake

A subset of membrane systems was chosen, for which the PEG1500 weight percentage was kept constant for both water uptake and ionic liquid uptake (ILU) experiments. In Table 3 and Table 4, water uptake and ionic liquid uptake results based on an average of three different membrane systems can be seen, respectively. These experiments were repeated for each SMC membrane group with four samples, testing each sample four times (one membrane group × four samples × four measurements). 

As can be seen in Table 3 and Table 4, uptake behaviors were different. ILU increased when the dianhydride composition changed to incorporate more 6FDA and even higher when only 6FDA was used. All the membranes’ ILU absorptions, when they were doped for 24 h, were in the same order of magnitude. In contrast, water uptake results decreased with more fluorinated dianhydride incorporation and were even lower when only 6FDA was used in the membrane. This lower water uptake could be due to the hydrophobicity of fluorinated molecules. 

#### 3.2.4. Conductivity

The ionic conductivity values were measured to establish the influence of polyimide structure on this key transport property. The conductivity results are given in Table 5. In addition, to see the effect of the thermal heating process on the undoped and EAN-doped membranes, conductivity measurements at room temperature using an Autolab brand instrument were also completed with changing imide compositions. The thermal annealing process was conducted for 1 h on a hot plate held at a constant temperature of 60 °C. These results given in Table 6 were acquired via repeating the tests for each SMC membrane group, using four samples and testing each sample four times (one membrane group × four samples × four measurements). 

From Table 5, SBC membranes, synthesized using equal amounts of two different functionalized dianhydrides, resulted in a higher conductivity value when compared with SBC membranes containing only one functionalized dianhydride at the same conditions of 60 °C and 70% RH. 

As can be seen from Table 6, there are various important results. First, thermal annealing of undoped membranes significantly increases conductivity by a factor of 10 in all cases. Even though the conductivities of the undoped membranes were very small, it was worth investigating to see the effect of thermal heating in the membranes. Second, when SBC membranes were doped with IL, the conductivity values significantly increased. This positive effect can be understandable when molecules have high ILU values presented in Table 4. Even though ILU values were in the same order of magnitude, with decreasing 6FDA content in the SBC membranes, the conductivity values were decreased almost in all cases. Third, thermal annealing had a further effect on the doped membranes in favor of conductivity. The thermal annealing process was seen to increase the conductivities for PEG-PI SBC membranes with PEG contents higher than 26.2 wt% by a factor of two to five times [39]. The same positive effect is present for all types of SBC membranes studied, where the conductivities were increased by a factor of two to seven. 

Readers are encouraged to visit the supplementary file to see how the resistivity values of these SBC membranes were affected due to ILU and thermal annealing. In summary, all the doped membranes showed increased conductivity after the 1 h thermal annealing process. 

#### 3.2.5. Tensile Test

**Figure 4:**
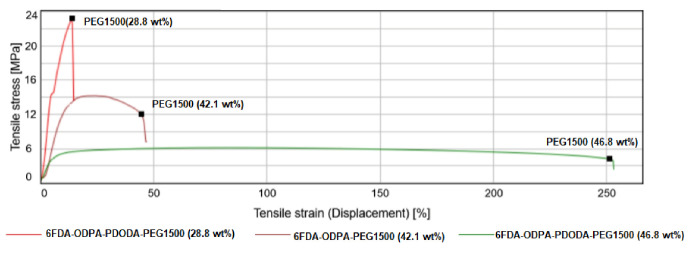
Stress–strain curves obtained from the tensile test for not thermally treated SBC membranes with changing PEG1500 content.

**Figure 5:**
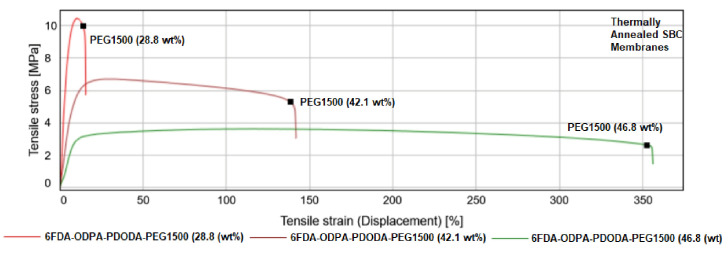
Stress–strain curves obtained from the tensile test for 1 h thermally treated SBC membranes with changing PEG1500 content.

Mechanical properties depend on the composition of the SBC membranes. Thus, several different mechanical property measurements of the membrane materials were studied when changing the PEG concentration and changing the imide composition. The most common polymer tests are tensile tests. A typical tensile test for undoped membranes untreated with heat and thermally annealed are shown in Figure 4 and Figure 5, respectively. A variety of information can be obtained from the tensile test; however, two properties, the tensile stress at maximum force and Young’s modulus, are of interest. A clear transition from brittle to ductile in mechanical properties was observed when the incorporation of PEG concentration was increased.

A clear transition from brittle to ductile mechanical properties is observed when the incorporation of PEG concentration was increased in all cases (Figure 4 and Figure 5). The thermal annealing process made the membranes more flexible, especially with a PEG content higher than 28.8 wt%. The most important observation can be made by analyzing the tensile stress at maximum force and the Young’s modulus values, as presented in Table 7. 

The effect of combining both 6FDA and ODPA dianhydrides resulted in much better mechanical properties. Furthermore, even when the samples were subjected to thermal annealing, they were stiff enough to handle, with the membranes with a PEG content lower than 46.8 wt% having a Young’s modulus around 100 MPa. 

**Figure 6:**
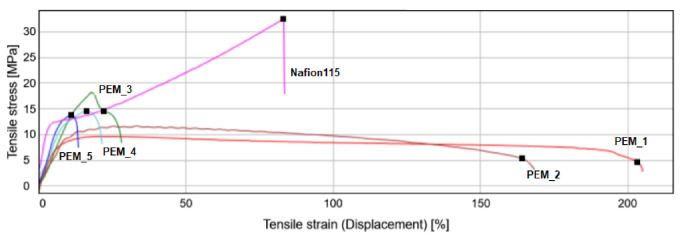
Stress–strain curves were obtained from the tensile test for the PI-PEG SBC membranes with changing dianhydride composition and constant PEG content of 42.1 wt% and Nafion 115, where Nafion 115 is used for comparison purposes.

Figure 6 shows the stress–strain curves for the SBC membranes with a changing imide composition and with a constant PEG concentration of 42.1 wt%, and Table 8 summarizes the two properties of the corresponding membranes. 

A clear transition in mechanical properties with changing incorporated dianhydrides can also be observed from the stress–strain curve. Nafion 115 was also tested for the same testing conditions. Both membranes showed strain hardening after passing the yield point, beyond which this effect reduces ductility and increases the chances of brittle failure [12]. By contrast, the fluorinated membrane (PEM_1) and the membrane containing both dianhydrides (PEM_2) showed higher elongation at break values possessing a more ductile type of behavior.

On the other hand, tensile at break values showed an interesting pattern. The value increased with increasing ODPA content up to 15 wt% and then started to decrease for the higher content of 15 wt%. 

In summary, the effect of mixing oxygenated and fluorinate dianhydrides can be seen in Figure 6. Clear transitions in mechanical property with increasing amounts of oxygenated dianhydride (ODPA) segments from ductile to brittle are observed. However, all the membranes synthesized were stiff enough to be handled, having Young’s modulus values comparable to Nafion (Table 8). It is shown that better materials were able to be synthesized in this study. 

## 4. Discussion

The results presented in this study show how the properties of these SBC membranes were affected by changing PEG concentration, changing the imide composition, and applying the thermal annealing process. There are few data available concerning both conductivities and mechanical properties as a function of temperature for PEG containing PIs with containing different dianhydride compositions. 

However, recent studies by Coletta et al. and Woo et al. focused on developing new PEMs with only one type of dianhydride, either using fluorination or oxygenation to synthesize PI-PEG SBC membranes [13,28,34,39]. The performance of these membranes surpasses Nafion 115 in terms of conductivity. They showed that these PI-PEG SBC membranes can function in lower humidity, exhibited good conductivity, and were shown to have stability in evaluated temperatures. Thus, it is worth analyzing the TGA results in more detail. There are slight differences in the thermal stabilities seen in the TGA results (Figure 1, Figure 2 and Figure 3). These small changes result from the difference in the PEG content within the system (Figure 1 and Figure 2) or the presence of more than one type of dianhydride (Figure 3). These slight changes are thought to result from differences in the molecular structure of the membranes. As the PEG content increases, the flexibility of the membranes increases. This behavior of the membranes was also observed in the tensile test results given in the previous section. In contrast, decreases in the PEG content enhance the proportion of stiff aromatic chains existing in the system, resulting in a slightly more rigid structure overall. Additionally, analyzing both the mechanical test results given in this study and in Coletta’s et al. previous works [13,28], the imidization process is thought to have a higher yield when a greater PEG content is incorporated. As shown by the tensile test results, a greater PEG content provides more flexibility in the membrane systems.

The effect of incorporating higher PEG1500 concentrations can be also seen from the FTIR spectrum, where the system with lower PEG content had a smaller height indicating less complete imidization reaction (Supplementary Figures S2–S5). In short, the ease of movement at the molecular level influences the imidization process, which causes slight weight loss differences at lower temperatures up to 300 °C. However, these membranes are all promising and stable enough to be used in applications with working conditions either above 100 °C or at low humidity.

In order to have an environment where ions can move more freely, an amorphous structure is needed. To achieve this kind of structure, PEG crystallization needed to be retarded [34,50,51,52,53]. Endotherms related to PEG crystallization are seen at around 40 to 53 °C for pure PEG reactants [34,39,54]. By contrast, it was shown that systems with shorter PEG chains and covalent bonds with aromatic monomers surrounding PEG segments are stronger, more stable and flexible. They yielded homogenous membranes with no evidence of PEG crystallinity, as also noted in the previous works [13,28,34,39]. Moreover, it was shown by Woo et al. via DSC analysis of EAN-doped membranes that PI-PEG membranes showed two different phases; one is rubbery (PEG-EAN domains) and the other glassy (PI-EAN domains), where no evidence of crystallinity was observed for the 6FDA-PDODA-PEG1500 SBC membrane studied. The same amorphous morphology which was documented by others was also achieved for all the membranes synthesized in this work [13,28,34,39].

It is worth mentioning here that the reaction between dianhydrides and diamines is required to have optimal conditions [54]. It was also shown that the polymer synthesized in DMAc medium had more rigidity than the 1-methyl-2-pyrrolidinone (NMP) [13]. This can be explained by the structure of DMAc having a less aromatic nature than other aprotic dipolar solvents, causing a less miscible environment for aromatic polyimide to form more structural order during the thermal imidization step [34,55,56,57,58,59,60,61]. Thus, to obtain the right environment, *N*,*N*-dimethylacetamide (DMAc) was chosen as a solvent because of its dipolar aprotic nature.

The structural changes investigated by Coletta et al. via SAXS experiments can be used to explain why ILU increases when more fluorinated dianhydride is incorporated in the PEG-PI SBC membranes. In a recent investigation, switching the dianhydrides from ODPA to 6FDA resulted in more randomly distributed PEG domains, whereas ODPA containing polyimide showed both PEG domains and aromatic polyimide order. Since PEG1500 is known to be readily soluble in EAN, more ILU should be observed for the systems with more PEG domains. The results presented in Table 4 confirm the findings presented by Coletta et al. [13,28,34], where ILU increased with the presence of 6FDA. Thus, it is thought that 6FDA’s incorporation into the PEG-PI SBC membranes resulted in a molecular structure where more microphase separation occurred, which generated microchannels that are interconnected. The generated microchannels in the PEG-PI system validate the abovementioned results where higher ILU values, more flexibility, and higher conductivities were observed. On the other hand, when the incorporation of 6FDA was decreased, the flexibility of the membranes also decreased, but since molecular structural domains (PEG and aromatic structural domains) still remained in the membranes, ILU and good conductivity values were observed. 

It is also believed that more randomly distributed PEG-rich segments cause higher mobility in the structure. Since the mobility is higher, it creates an opportunity for more ionic liquid uptake. This proposed morphology is supported by the studies performed by creating microchannels, as proposed by Coletta et al. and Woo et al. [13,28,34,39]. In the oxygenated system, as is shown in the mechanical test results, the molecular structure is more rigid than the fluorinated system. Due to its structural rigidity, a lesser amount of IL was incorporated when compared to the fluorinated system. On the other hand, due to the flexibility gained by the fluorinated dianhydride entity, the ILU was increased, and the results were almost of the same order of magnitude for 6FDA containing SBC membranes.

In contrast, the WU for the oxygenated membrane system was almost twice that of the fluorinated system (Table 3). This may be due to the hydrophobicity of those fluorinated groups in the SBC membranes contouring the PEG domains and retarding the sorption of water. More detailed studies are needed to show the water sorption and its kinetics with changes in the temperature to fully understand this phenomenon. It should be noted that PEG-containing PI membranes can operate under lower humidity levels and are not that affected by the need for hydration. In contrast, hydration and water management are very critical for Nafion and its derivatives to operate effectively [8].

As can be seen from Table 6, incorporating two different dianhydrides with different functional bridging groups validates this study by obtaining a higher conductivity. 6FDA+ODPA-PDODA-PEG1500 has the highest conductivity in this group, comparable to that with Nafion 115, which has an average conductivity of 39.4 mS/cm measured at 60 °C with 70% RH doped in 20% phosphoric acid solution examined by Woo et al. [39]. Most importantly, when the corresponding materials’ stress–strain curves (Figure 4and Figure 5) and mechanical properties listed in Table 7 are analyzed, it can be seen that better materials were successfully synthesized. 

The membranes became less stiff with increasing fluorinated dianhydride composition (Figure 6). Flexibility seems to be the key property of an effective membrane, so we wish to emphasize its role. Flexibility in the material may enable an easier structural change, creating interconnected networking microchannels swollen with EAN. This is also in line with ILU values presented by us and Woo et al. [39]. We suggest that mixing two different dianhydrides creates more well-defined PEG1500-rich domains in the structure, which impart rubbery characteristics (interconnected networking structures), and polyimide-rich domains, causing strength (mechanical integrity). Due to these structural morphologies within the material, higher conductivity was achieved.

In the same recent study, the effects of the thermal annealing process on the structure and the conductivity of PI-PEG SBC membranes were also presented. The study established that the conductivity increased and the molecular structure changed so as to favor more EAN uptake [39]. The highest conductivity was found to be ~210 mS/cm for the fluorinated SBC membrane with 42.1 wt% PEG1500. Therefore, it seems that these positive effects could also be seen in the 6FDA+ODPA-PDODA-PEG1500 SBC membrane family. However, it was expressed by Woo et al. that the membranes possessing conductivity values higher than 150 mScm^−1^ showed very weak mechanical strengths, becoming very soft and easily torn. The mechanical tests were not performed for all the doped membranes in this study; however, physical observations are such that the membranes with equally mixed imide compositions (PEM_3) were less swollen with no change in the width of the membrane and were still stiff enough to be handled. 

All measurements were repeated for each of the films after 1 h thermal annealing. As can be seen from Table 6, there are various important outcomes. First, thermal annealing of undoped membranes significantly increases conductivity by a factor of ten. This positive effect of the thermal annealing process may be due to the fact that PEG-rich domains create a flexible environment, whereas polymer chains tend to create some stiffness. This behavior was indicated by a broad shoulder in the SAXS results on thermally annealed undoped membranes [39]. It was also shown that fluorinated polyimides are bulkier and relatively flexible relative to nonfluorinated PIs, but the fluorinated systems do not possess as much regularity as found for unbulky oxygenated polyimides [62,63,64,65]. The effect of thermal annealing on the PI-PEG membrane with only fluorinated dianhydride was slightly less than the effect seen for the PI-PEG membrane consisting of only oxygenated dianhydride. This is believed to be due to the oxygenated PI-PEG systems being stiffer. However, upon heating, PEG segments became more mobile and tended to change their spatial correlations by creating PEG domains through reduced miscibility. In short, it is believed that via thermal annealing the mobility at the molecular level is reduced, thus decreasing the resistivity of the bulk membranes. The reduction in resistivity is observed for all the SBC membranes. 

Upon doping, the conductivity of the membrane increases [1]. It was also shown in many studies in the literature that PI-based PEMs increased ion-transfer in the presence of an IL [1,28,34,39,40,41]. Since the synthesized membranes showed good ILU values, all the doped membranes showed increased conductivity. IL being present in the molecular structure is believed to play an important role for the electron transfer via connecting the generated microchannels, creating easier paths for the transport of the ions. Another method was to increase the connection of these microchannels, and thus thermal annealing, as proposed by Woo et al. was conducted [39].

Thermal annealing on a hot plate for undoped and doped membranes showed much improved results, with the conductivity exceeding expectations. More interestingly, when the films are subject to heating, the spatial correlation of the PEG+EAN domains increases [39]. The structural change of PI-PEG membranes containing fluorinated dianhydride, oxygenated diamine, and 42.1 wt% of PEG was studied extensively to see the effect of IL doping and as well as thermal annealing by Woo et al. [39]. In the study, it was shown that in the presence of IL and applying a thermal annealing process, PEG-EAN domains became more defined and the membranes showed the same pattern of bicontinuous structured materials that were given in the literature [39]. So, the effect of thermal annealing on the membrane is seen as an increase in IL uptake by the membrane. A greater IL presence in the membrane means higher ion-transfer. If this evaluation proves to be correct, then one would measure even higher conductivity values when the PEM films were subjected to heat. The results presented in Table 6 confirm this evaluation, where higher conductivity results were successfully measured. These results found for conductivity values also give support to the evaluations of the SAXS experiments presented in the literature, where more interconnected segmented bicontinuous structures were seen in membranes when they were doped in an IL or thermally annealed [30,39,50,61]. In short, these findings also confirm that the thermal annealing of free-standing films, even at 60 °C, plays a key role in structural change via the increased mobility of the molecules, where it contributes to the increased bicontinuous phase-separated structure, resulting in higher ILU and a higher conductivity property, for which most of the values are a factor of three or higher greater than the Nafion 115 value reported earlier by Woo et al. [39]. 

## 5. Materials and Methods

### 5.1. Materials

4,4′-oxydiphthalic anhydride (ODPA, an aromatic dianhydride), 4,4′-(hexafluoroisopropylidene) diphthalic anhydride (6FDA, an aromatic dianhydride), 4,4′-(1,3-phenylenedioxy) dianiline (PDODA, an aromatic diamine), and poly(ethylene glycol) bis(3-aminopropyl) terminated PEG1500 functionalized with amine groups on both ends (numbers indicating the molecular weights of the PEG diamines) were used to build the membrane copolymer matrix. 6FDA, ODPA, PDODA, and PEG1500 were purchased from Sigma-Aldrich (St. Louis, MO, USA). The ionic liquid ethylammonium nitrate (EAN, >97%), and propylammonium nitrate (PAN, >97%) were purchased from Iolitec (Heilbronn, Germany). Solvent reagent, *N*,*N*-dimethylacetamide (DMAc) was purchased from Sigma Aldrich. All the materials were used as received.

### 5.2. Synthesis

**Figure 7:**
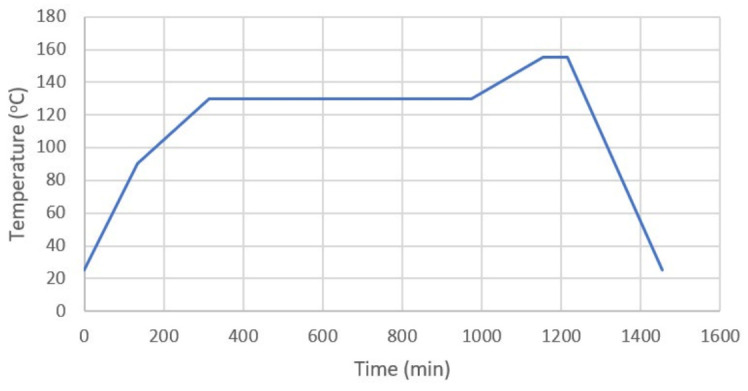
Heating protocol used for the synthesis of the membranes.

**Figure 8:**
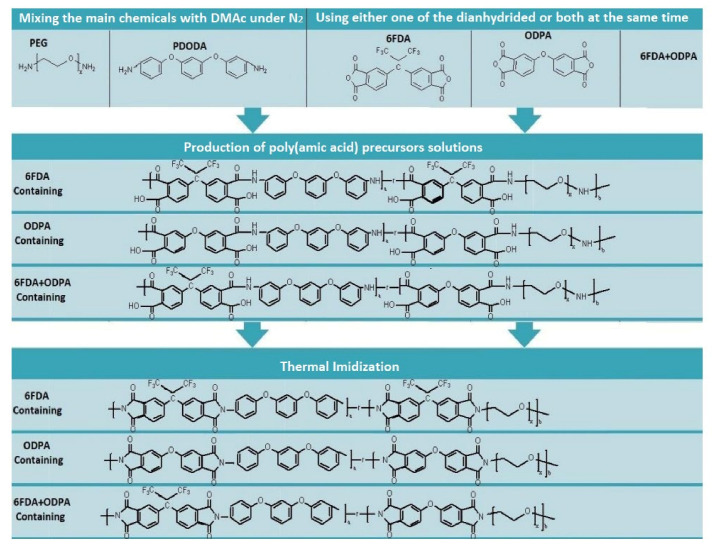
Schematic representation of two-step condensation polymerization and chemical structures of the synthesized polyimides.

A two-step condensation polymerization synthesis methodology was conducted to form the polyimide-PEG random copolymers. Initially, PEG1500 and PDODA were placed in a three-necked flask. Then, approximately 40 mL of DMAc was poured in, and the mixture was stirred and gently heated to around 45 °C in a nitrogen environment until all solids were dissolved. After cooling to room temperature, either one of the aromatic dianhydrides or both together were placed in a three-neck flask in solid form for approximately an hour. The solution was stirred in a nitrogen environment for 24 h to evaluate the poly(amic acids) (PAA) (see Figure 7). After evaluating the PAA solutions, the content was poured into a Teflon-lined Petri dish for thermal imidization. The heating protocol for this step is given in Figure 7, and the summary of the synthesis process is summarized in Figure 8, which shows the key chemical structures of each main step.

### 5.3. Characterization

#### 5.3.1. FTIR

FTIR measurements were taken using a Nicolet iS50 FT/IR Spectrometer at room temperature in attenuated total reflection (ATR) mode. For FTIR analysis, a small amount of every synthesized PAA sample solution and their corresponding imidized SBC membranes were cast on a glass slide. The FTIR results were used to confirm the success of the synthesis and imidization process.

#### 5.3.2. TG

Using a TA Instrument Q500, membrane samples weighing between 8 and 12 mg were loaded into alumina pans for testing. The samples were warmed from 25 to 600 °C at 10 °C/min and the results were then analyzed.

#### 5.3.3. DSC

For DSC analysis, samples weighing between 8 and 12 mg were loaded into aluminum pans. The samples were treated according to the thermal protocol previously reported by Coletta et al. [13]: equilibrate at 20 °C, ramp at 5 °C/min up to 120 °C, hold at 120 °C for 2 min, ramp at 5 °C/min to 20 °C, hold at 20 °C for 2 min, and then repeat the cycle two more times. 

### 5.4. Ionic Liquid and Water Uptake

In these experiments, free-standing films were cut into approximately 5 ± 2 cm × 2 ± 0.4 cm size and weighed before placing into either water or EAN at room temperature for 24 h. Subsequently, the films were removed and reweighed after drying the excess liquids on the surface of the membranes with kim wipes. 

### 5.5. Conductivity

To see the effect of temperature on the conductivity and performance of the synthesized SBC membranes, a BT 552 Bekktech conductivity analyzer and a Gamry Instruments Reference 600 Potentiostat-Galvanostat-ZRA were used. A frequency range of 200,000 to 0.1 Hz with an amplitude of 10 mV was chosen to perform the AC impedance measurement, for 6 points per decade. Analysis was carried out with a nitrogen atmosphere at 70% RH at 60 and 70 °C. Three different films were prepared for each set of experiments, so the values represent the averaged conductivity. The individual film measurement values were based on an average of five measurements per single temperature on the same film.

To see the effect of changing the ionic liquid and thermal annealing process on the conductivity of membranes, measurements are taken using Autolab PGSTAT204 potentiostat/galvanostat. The AC impedance was measured under an ambient atmosphere at 25 °C over a frequency range of 200,000 to 0.1 Hz, with an amplitude of 10 mV, for 10 points per decade. Three different films were prepared for each set of experiments. Then, values were acquired for all prepared films, taking three measurements per single temperature on the same film, so the values represent the average conductivities for six different conditions tested. 

### 5.6. Tensile

Mechanical strengths of the SBC membranes, thermally not annealed and thermally annealed, were tested using an Instron 5500 series system with a constant strain rate of 5 mm/s. Rectangular-shaped membrane strips of around 2 ± 0.5 cm by 10 ± 5 cm were used in these tensile tests.

## 6. Conclusions

In this study, we evaluated the impact of the incorporation of multi-dianhydrides and sought deeper insight into the effect of thermal annealing on the membrane’s conductivities. The measured values shown here provide a further validation of the principle that a structural transition in the segmented block copolymer structure (where EAN-PEG domains are connected through a web of microchannels) enhances conductivity. The values measured for oxygenated systems were found to be around three times higher than Nafion 115. The fluorinated system and the multi-dianhydride system with thermal annealing before conductivity measurements had the highest conductivity values, eight times higher than Nafion 115. Especially, materials with better mechanical properties were synthesized via thermal annealing, where increased interconnected bicontinuous phase-separated structures were obtained.

## Figures and Tables

**Table 1:** SBC membrane systems with varying PEG1500 wt%.

PEG-PI Systems.	PEG wt%	6FDA wt% + ODPA wt%	PDODA wt%
6FDA-PDODA-PEG1500	28.8	48 + 0	23.2
	42.1	30 + 0	27.9
	46.8	38 + 0	15.2
ODPA-PDODA-PEG1500	28.8	0 + 48	23.2
	42.1	0 + 30	27.9
	46.8	0 + 38	15.2
6FDA+ODPA-PDODA-PEG1500 (50/50 wt% of dianhydrides)	28.8	24 + 24	23.2
	42.1	15 + 15	27.9
	46.8	19 + 19	15.2

**Table 2:** SBC membrane systems with varying wt% of dianhydrides.

	6FDA wt% (in All Materials)	ODPA wt% (in All Materials)
6FDA + ODPA-PDODA-PEG1500	30	0
	22.5	7.5
	19	19
	7.5	22.5
	0	30

**Table 3:** WU values.

Material (PEG1500: 42.1 wt%)	Liquid (at 25 °C)	wU (%)
6FDA-PDODA-PEG1500	Water	22 ± 10
ODPA-PDODA-PEG1500	Water	45 ± 12
6FDA + ODPA-PDODA-PEG1500	Water	42 ± 10

**Table 4:** ILU values.

Material (PEG1500: 42.1 wt%)	IL (at 25 °C)	ILU (%)
6FDA-PDODA-PEG1500	EAN	106 ± 14
ODPA-PDODA-PEG1500	EAN	80 ± 9
6FDA + ODPA-PDODA-PEG1500	EAN	89 ± 11

**Table 5:** Conductivity measurements of the EAN-doped SBC membranes (values are for the SBC membranes that are not thermally annealed. For detailed calculations please see Supplementary Table S1).

Material	Conductivity (mS/cm) (at 60 °C and RH 70%)
6FDA-PDODA-PEG1500 ^a^ (PEG1500: 50 wt%)	80 ± 15
ODPA-PDODA-PEG1500 ^b^ (PEG1500: 50 wt%)	16 ± 4
6FDA-PDODA-PEG1500 ^c^ (PEG1500: 42.1 wt%)	60 ± 20
6FDA + ODPA,-PDODA-PEG1500 ^d^ (PEG1500: 42.1 wt%)	84 ± 17

^a^ From ref. [13]. ^b^ From ref. [34]. ^c^ From ref. [39]. ^d^ Detailed calculation of the value is given in Supplementary Table S1.

**Table 6:** Comparison of conductivity of membranes without and with thermal annealing.

SBC Membranes	No Thermal Annealing Processes		1 h Thermal Annealing Process	
	Conductivities of Undoped Membrane Films (mS/cm)	Conductivities of Doped Membrane Films in EAN Ionic Liquid (mS/cm)	Conductivities of Undoped Membrane Films (mS/cm)	Conductivities of Doped Membrane Films in EAN Ionic Liquid (mS/cm)
6FDA (30 wt%)- PDODA-PEG1500	34 × 10^–4^	81	20 × 10^–3^	334
6FDA (22.5 wt%)-ODPA (7.5 wt%) -PDODA- PEG1500	49 × 10^–4^	73	30 × 10^–3^	152
6FDA (15 wt%)-ODPA (15 wt%)-PDODA-PEG1500	8 × 10^–4^	57	15 × 10^–3^	307
6FDA (7.5 wt%)-ODPA (22.5 wt%) -PDODAPEG1500	4 × 10^–4^	42	6 × 10^–3^	285
ODPA (30 wt%)-PDODA-PEG1500	6 × 10^–4^	23	22 × 10^–3^	128

**Table 7:** Tensile stress at maximum force and the Young’s modulus of the SBC membranes with changing PEG content (Dianhydride entities are written in bold text).

	Thermally Untreated SBC Membranes		Thermally Annealed SBC Membranes	
	Tensile Stress at Maximum Force [MPa]	Young’s Modulus [MPa]	Tensile Stress at Maximum Force [MPa]	Young’s Modulus [MPa]
6FDA-PDODA-PEG1500 (28.8 wt%)	14.82	274.21	8.15	92.26
6FDA-PDODA-PEG1500 (42.1 wt%)	9.25	147.13	6.5	45.6
6FDA-PDODA-PEG1500 (46.8 wt%)	7.57	28.91	4.87	25.58
ODPA-PDODA-PEG1500 (28.8 wt%)	-	-	5.57	63.19
ODPA-PDODA-PEG1500 (42.1 wt%)	12.97	222.73	10.8	63.17
ODPA-PDODA-PEG1500 (46.8 wt%)	2.87	79.68	4.04	24.22
6FDA-ODPA-PDODA-PEG1500 (28.8 wt%)	23.21	289.02	10.45	155.89
6FDA-ODPA-PDODA-PEG1500 (42.1 wt%)	15.19	119.9	6.71	79.83
6FDA-ODPA-PDODA-PEG1500 (46.8 wt%)	5.11	67.03	3.63	41.78

**Table 8:** Tensile stress at maximum force and the Young’s modulus of the SBC membranes with changing dianhydride composition and constant PEG content of 42.1 wt% (dianhydride entities are written in bold text).

	Thermally Untreated SBC Membranes	
	Tensile Stress at Maximum Force [MPa]	Young’s Modulus [MPa]
6FDA (30 wt%)-ODPA (0 wt%)-PDODA-PEG1500	9.62	147.05
6FDA (22.5 wt%)-ODPA (7.5 wt%)-PDODA-PEG1500	11.67	166.92
6FDA (15 wt%)-ODPA (15 wt%)-PDODA-PEG1500	18.21	150.43
6FDA (7.5 wt%)-ODPA (22.5 wt%)-PDODA-PEG1500	14.53	152.18
6FDA (0 wt%)-ODPA (30 wt%)-PDODA-PEG1500	17.43	220.33
Nafion 115	32.46	288.94

## Data Availability

Not applicable.

## Supplementary Materials

The following are available online, Figure S1: a: FTIR spectra of the 6FDA-PDODA family of PEG containing polyimides with PEG1500 concentrations of 28.8 wt%,b: FTIR spectra of the 6FDA-PDODA family of PEG containing polyimides with PEG1500 concentrations of 42.1 wt%, c: FTIR spectra of the 6FDA-PDODA family of PEG containing polyimides with PEG1500 concentrations of 46.8 wt%; Figure S2: a: FTIR spectra of the ODPA-PDODA family of PEG containing polyimides with PEG1500 concentrations of 28.8wt%, b: FTIR spectra of the ODPA-PDODA family of PEG containing polyimides with PEG1500 concentrations of 42.1 wt%, c: FTIR spectra of the ODPA-PDODA family of PEG containing polyimides with PEG1500 concentrations of 46.8 wt%; Figure S3: a: FTIR spectra of the 6FDA+ODPA-PDODA family of PEG containing polyimides with PEG1500 concentrations of 28.8 wt%, b: FTIR spectra of the 6FDA+ODPA-PDODA family of PEG containing polyimides with PEG1500 concentrations of 42.1 wt%, c: FTIR spectra of the 6FDA+ODPA-PDODA family of PEG containing polyimides with PEG1500 concentrations of 46.8 wt%; Figure S4: a: Comparison of FTIR of 30 wt% 6FDA+ 0 wt% ODPA-PDODA-PEG1500 poly (amic acid) precursor and polyimide after imidization process (42.1 wt% of PEG1500) with given pick values, b: Height of the picks for 30 wt% 6FDA+ 0 wt% ODPA-PDODA-PEG1500 poly (amic acid) (42.1 wt% of PEG1500) precursor, c: Height of the picks for 30 wt% 6FDA+ 0 wt% ODPA-PDODA-PEG1500 polyimide after imidization process (42.1 wt% of PEG1500); Figure S5: a: Comparison of FTIR of 22.5 wt% 6FDA+ 7.5 wt% ODPA-PDODA-PEG1500 poly (amic acid) precursor and polyimide after imidization process (42.1 wt% of PEG1500) with given pick values, b: Height of the picks for 22.5 wt% 6FDA+ 7.5 wt% ODPA-PDODA-PEG1500 poly (amic acid) (42.1 wt% of PEG1500) precursor, c: Height of the picks for 22.5 wt% 6FDA+ 7.5 wt% ODPA-PDODA-PEG1500 polyimide after imidization process (42.1 wt% of PEG1500); Figure S6: a: Comparison of FTIR of 15 wt% 6FDA+ 15 wt% ODPA-PDODA-PEG1500 poly (amic acid) precursor and polyimide after imidization process (42.1 wt% of PEG1500) with given pick values, b: Height of the picks for 15 wt% 6FDA+ 15 wt% ODPA-PDODA-PEG1500 poly (amic acid) (42.1 wt% of PEG1500) precursor, c: Height of the picks for 15 wt% 6FDA+ 15 wt% ODPA-PDODA-PEG1500 polyimide after imidization process (42.1 wt% of PEG1500); Figure S7: a: Comparison of FTIR of 7.5 wt% 6FDA+ 22.5 wt% ODPA-PDODA-PEG1500 poly (amic acid) precursor and polyimide after imidization process (42.1 wt% of PEG1500) with given pick values, b: Height of the picks for 7.5 wt% 6FDA+ 22.5 wt% ODPA-PDODA-PEG1500 poly (amic acid) (42.1 wt% of PEG1500) precursor, c: Height of the picks for 7.5 wt% 6FDA+ 22.5 wt% ODPA-PDODA-PEG1500 polyimide after imidization process (42.1 wt% of PEG1500); Figure S8: a: Comparison of FTIR of 0 wt% 6FDA+ 30 wt% ODPA-PDODA-PEG1500 poly (amic acid) precursor and polyimide after imidization process (42.1 wt% of PEG1500) with given pick values, b: Height of the picks for 0 wt% 6FDA+ 30 wt% ODPA-PDODA-PEG1500 poly (amic acid) (42.1 wt% of PEG1500) precursor, c: Height of the picks for 0 wt% 6FDA+ 30 wt% ODPA-PDODA-PEG1500 polyimide after imidization process (42.1 wt% of PEG1500); Table S1: Detailed calculations of conductivity of the synthesized SBC membrane.
