# Supplementary material for: Influence of Mixed Imide Composition and Thermal Annealing on Ionic Liquid Uptake and Conductivity of Polyimide-Poly(ethylene glycol) Segmented Block Copolymer Membranes
Source: Molecules. 2021 Dec 9;26(24):7450. doi: 10.3390/molecules26247450 (PMC8705581; doi:10.3390/molecules26247450)
Supplement: Supplementary file 1 [file molecules-26-07450-s001.zip › molecules-1439567-supplementary.pdf]

Supplementary file

Influence of Mixed Imide Composition and Thermal Annealing on Ionic Liquid Uptake and Conductivity of Polyimide-Poly(ethylene glycol) Segmented Block Copolymer Membranes

Gokcen A. Ciftcioglu [1,2,*] and Curtis W. Frank [2]

[1]Department of Chemical Engineering, Marmara University, Istanbul, 34722, Turkey
[2]Department of Chemical Engineering, Stanford University, Stanford, CA 94305, USA

FTIR Figures

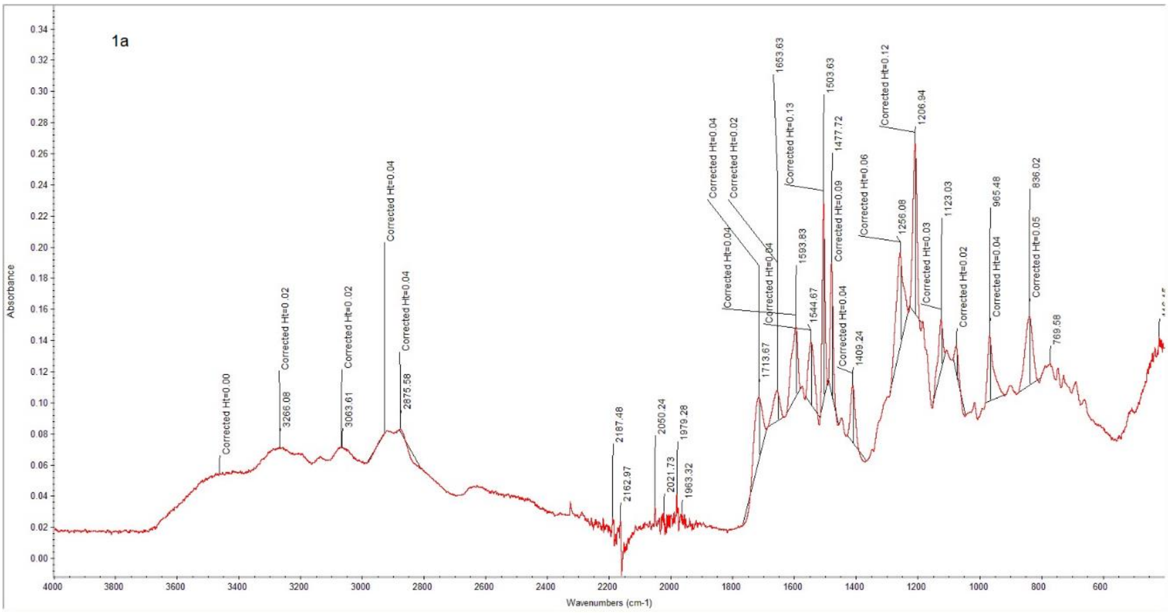

Figure S1a. FTIR spectra of the 6FDA-PDODA family of PEG containing polyimides with PEG1500 concentrations of 28.8 wt %.

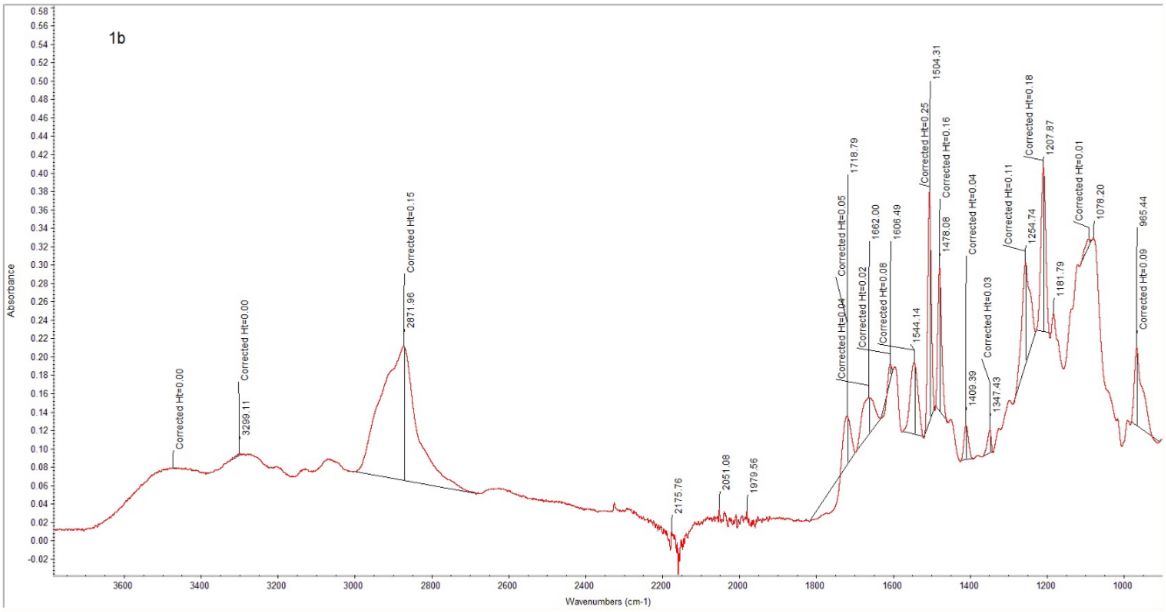

Figure S1b. FTIR spectra of the 6FDA-PDODA family of PEG containing polyimides with PEG1500 concentrations of 42.1 wt %.

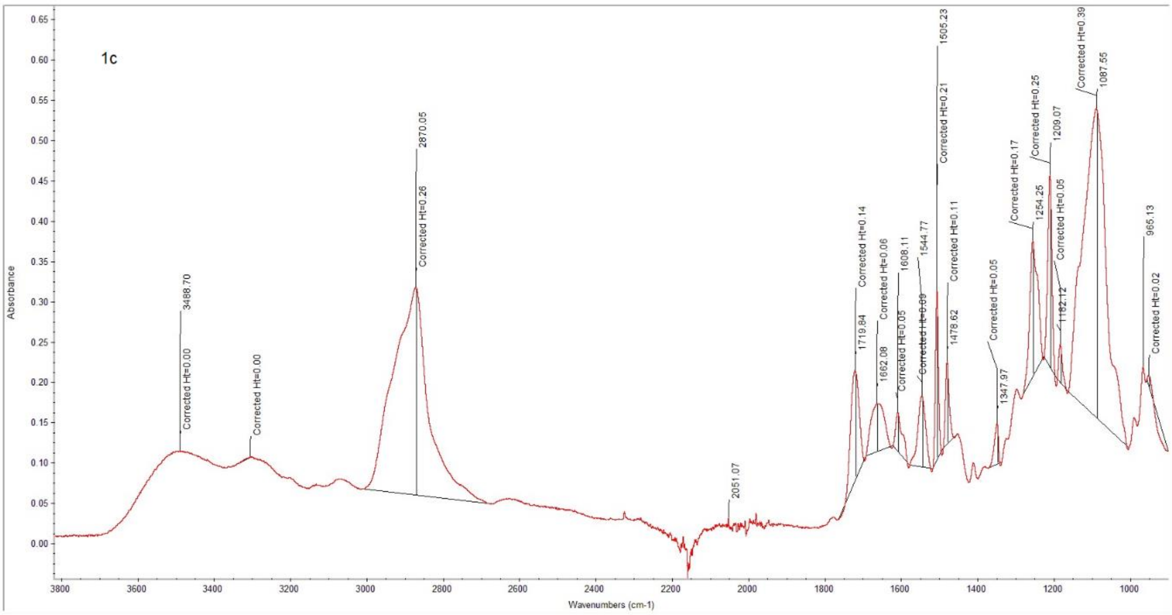

Figure S1c. FTIR spectra of the 6FDA-PDODA family of PEG containing polyimides with PEG1500 concentrations of 46.8 wt %.

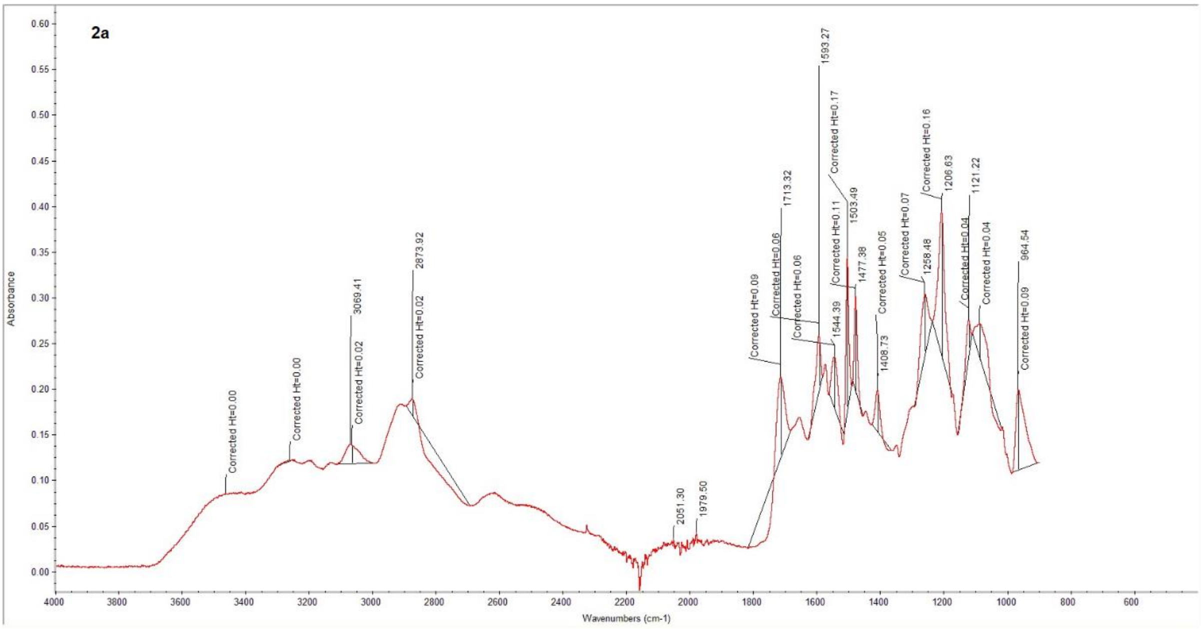

Figure S2a. FTIR spectra of the ODPA-PDODA family of PEG containing polyimides with PEG1500 concentrations of 28.8 wt %.

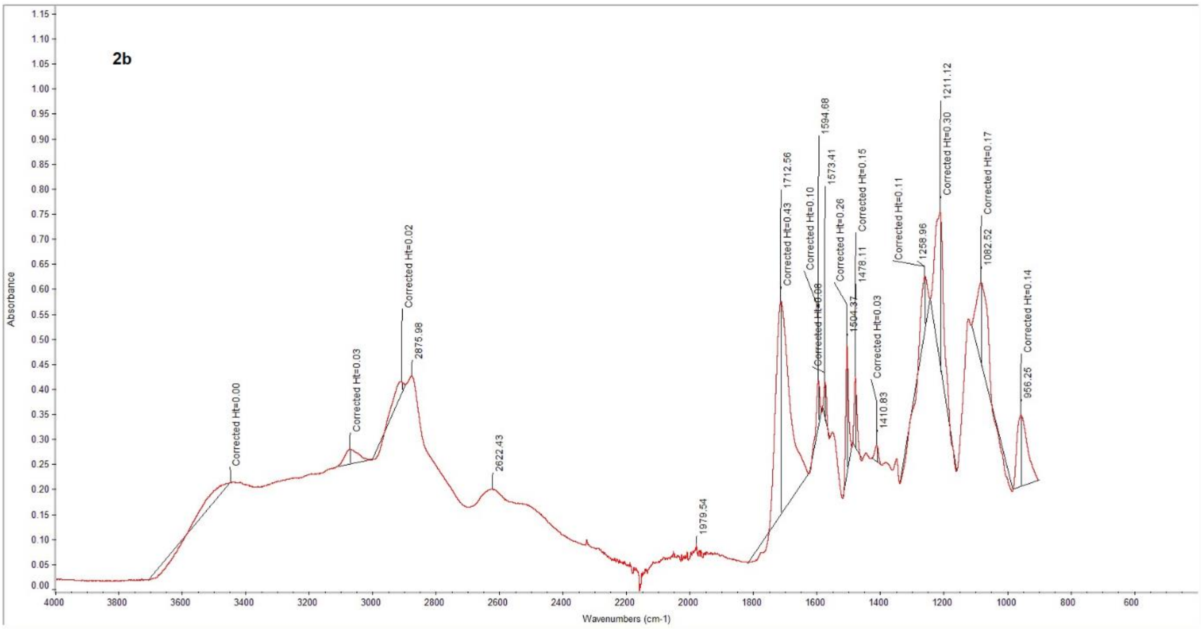

Figure S2b. FTIR spectra of the ODPA-PDODA family of PEG containing polyimides with PEG1500 concentrations of 42.1 wt %.

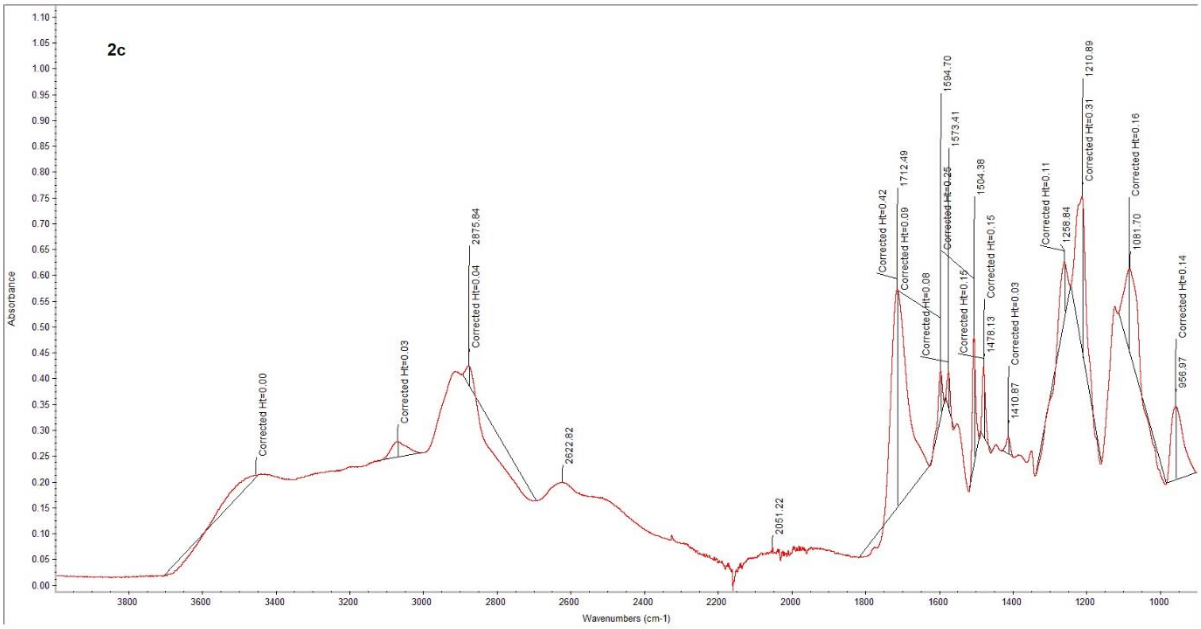

Figure S2c. FTIR spectra of the ODPA-PDODA family of PEG containing polyimides with PEG1500 concentrations of 46.8 wt %.

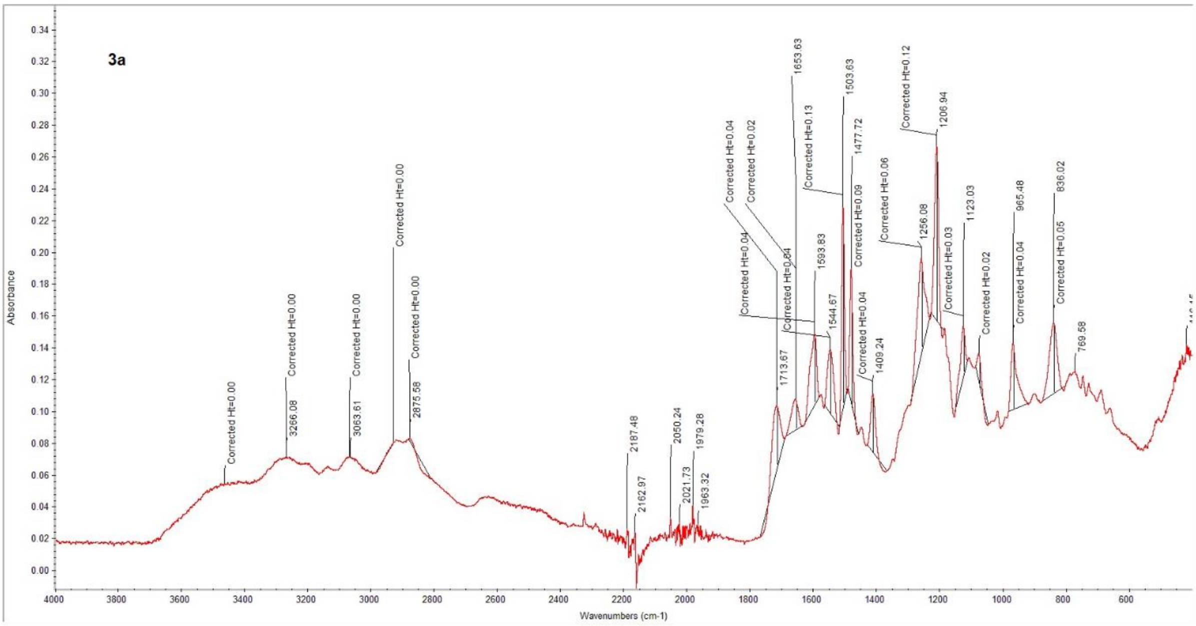

Figure S3a. FTIR spectra of the 6FDA+ODPA-PDODA family of PEG containing polyimides with PEG1500 concentrations of 28.8 wt %.

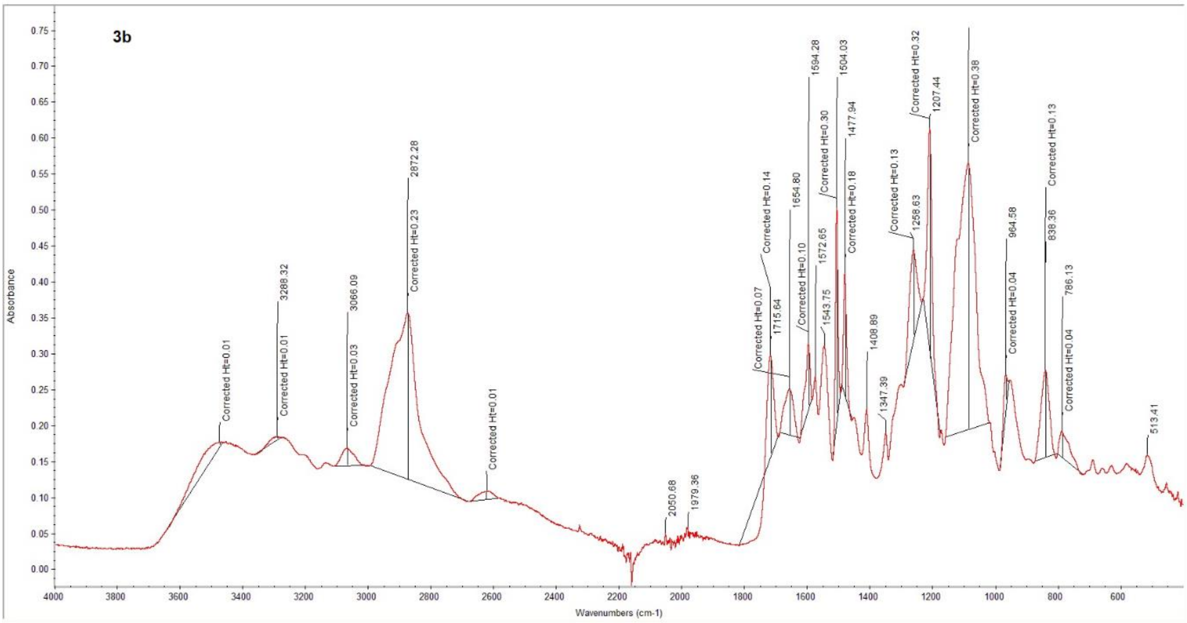

Figure S3b. FTIR spectra of the 6FDA+ODPA-PDODA family of PEG containing polyimides with PEG1500 concentrations of 42.1 wt %.

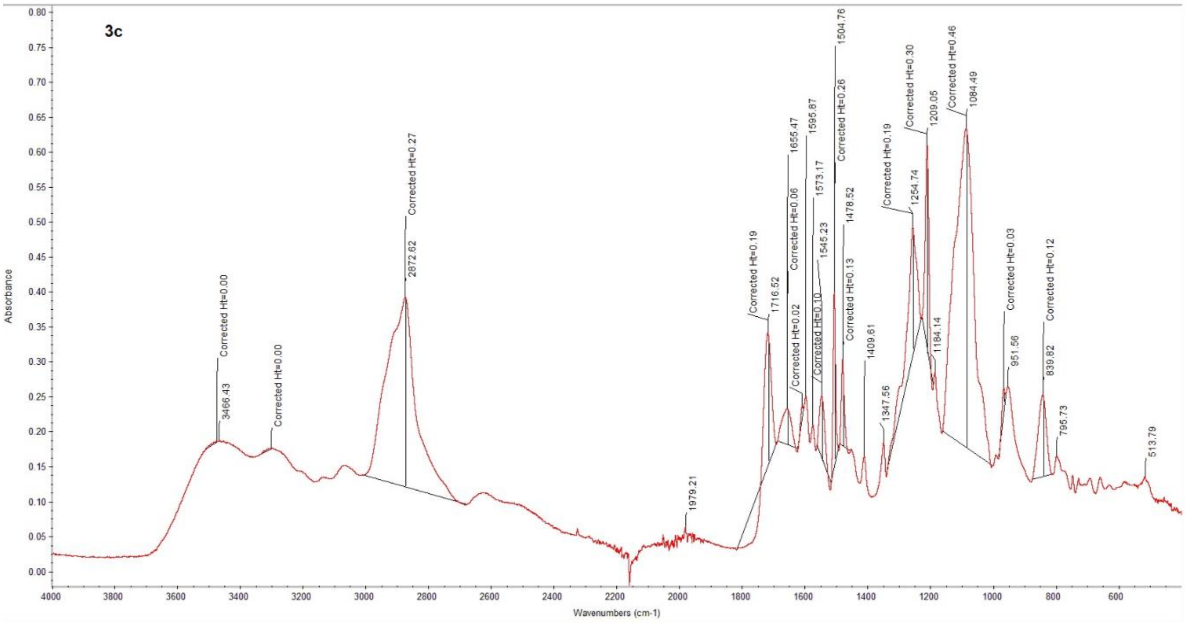

Figure S3c. FTIR spectra of the 6FDA+ODPA-PDODA family of PEG containing polyimides with PEG1500 concentrations of 46.8 wt %.

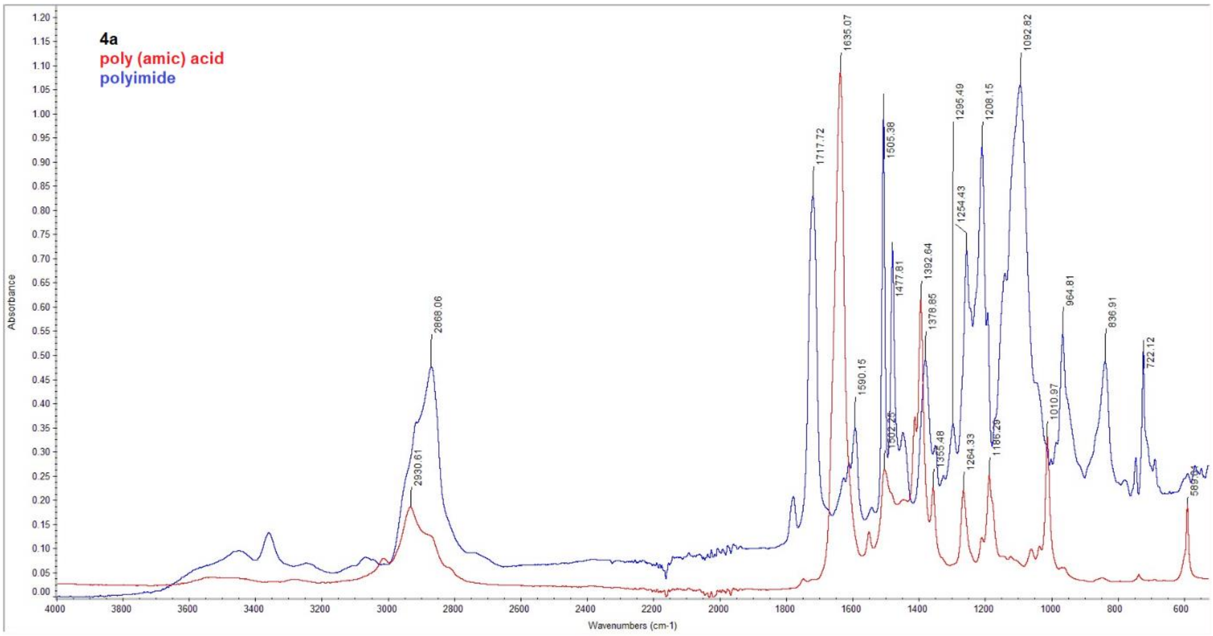

Figure S4a . Comparison of FTIR of 30 wt % 6FDA+ 0 wt% ODPA-PDODA-PEG1500 poly (amic acid) precursor and polyimide after imidization process (42.1 wt % of PEG1500) with given pick values.

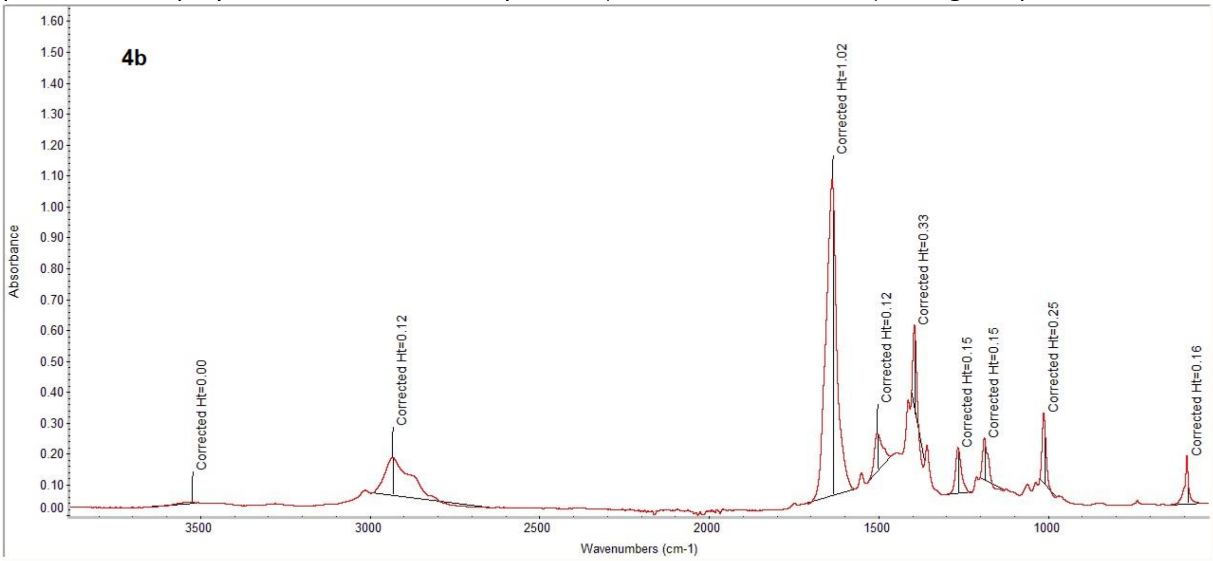

Figure S4b. Height of the picks for 30 wt % 6FDA+ 0 wt% ODPA-PDODA-PEG1500 poly (amic acid) (42.1 wt % of PEG1500) precursor

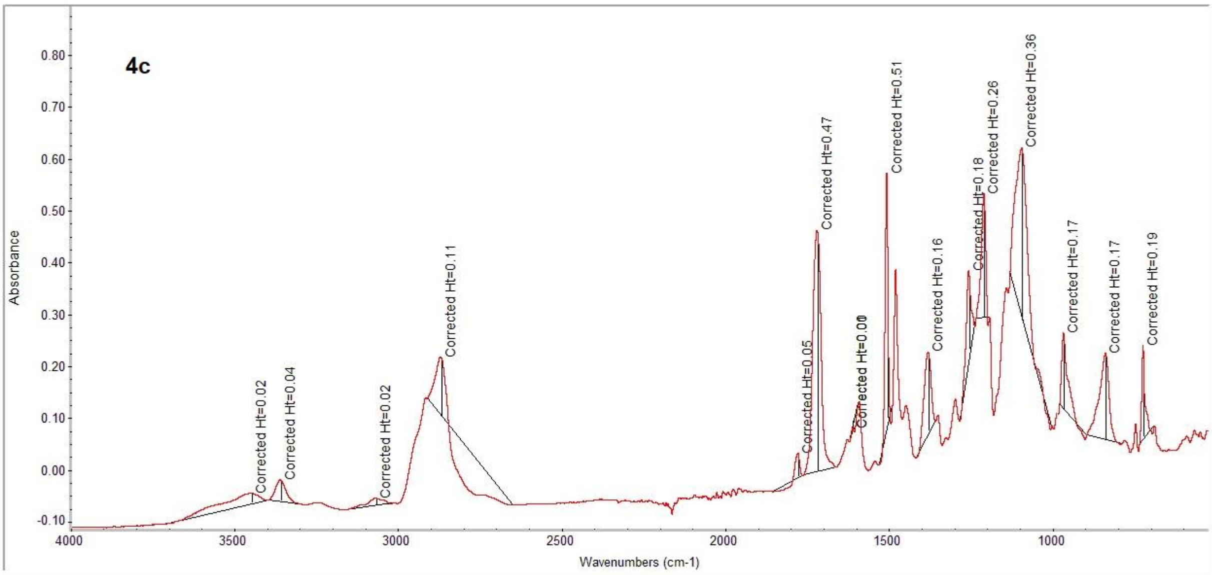

Figure S4c. Height of the picks for 30 wt % 6FDA+ 0 wt% ODPA-PDODA-PEG1500 polyimide after imidization process (42.1 wt % of PEG1500)

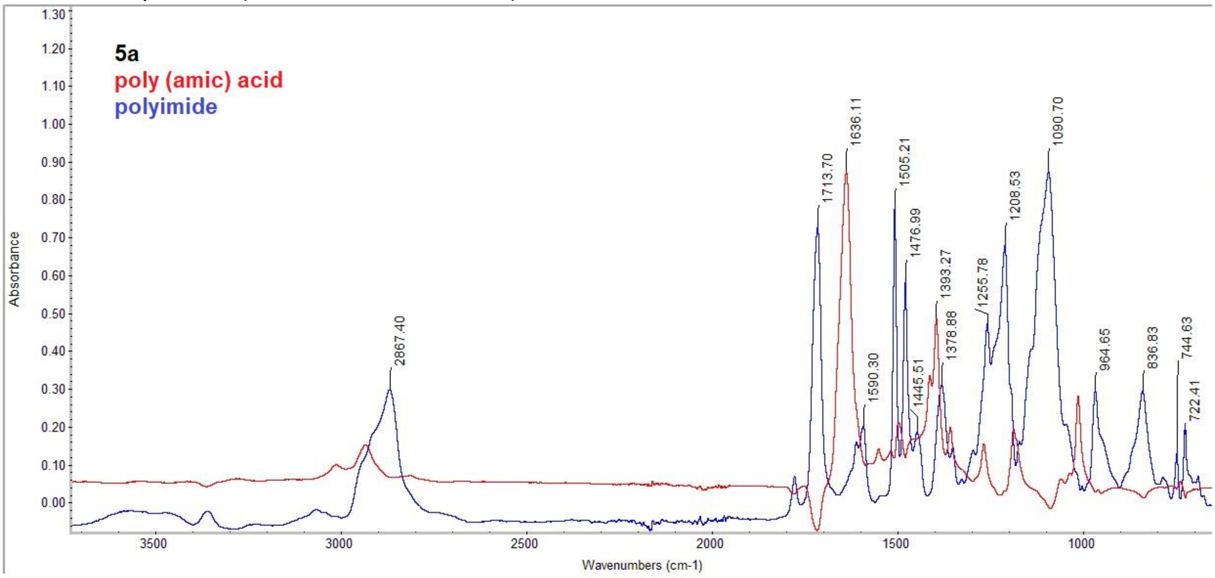

Figure S5a. Comparison of FTIR of 22.5 wt % 6FDA+ 7.5 wt% ODPA-PDODA-PEG1500 poly (amic acid) precursor and polyimide after imidization process (42.1 wt % of PEG1500) with given pick values.

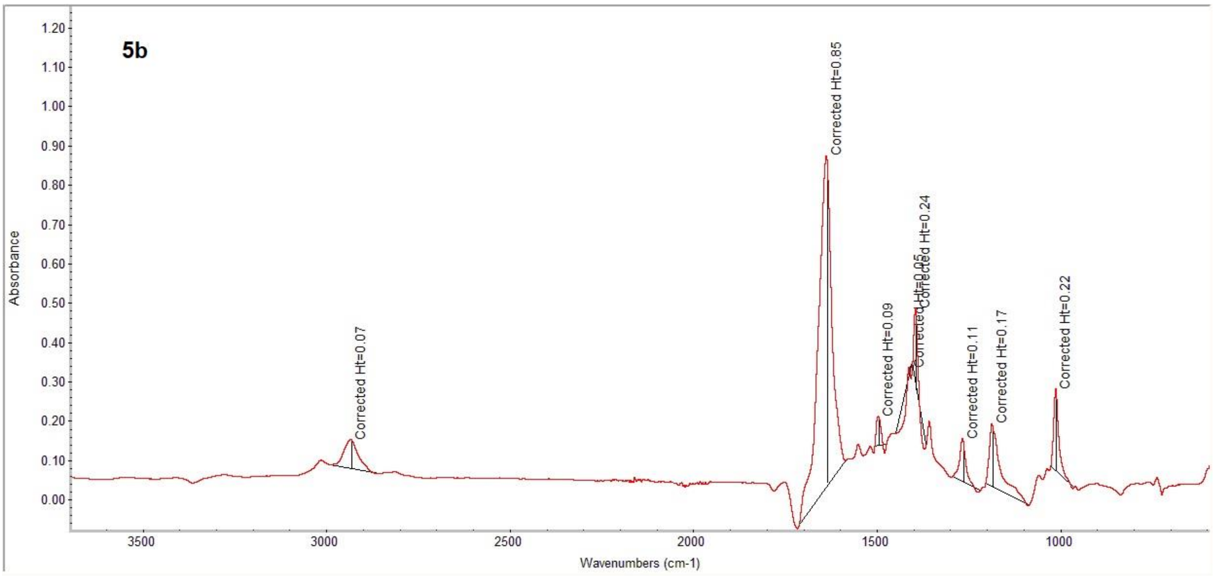

Figure S5b. Height of the picks for 22.5 wt % 6FDA+ 7.5 wt% ODPA-PDODA-PEG1500 poly (amic acid) (42.1 wt % of PEG1500) precursor

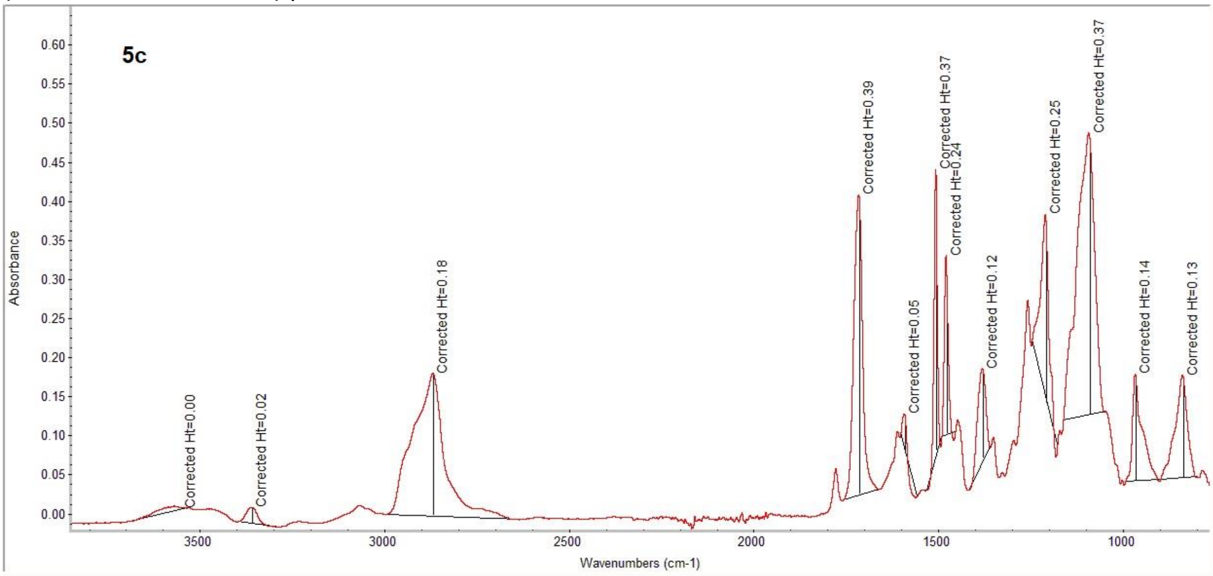

Figure S5c. Height of the picks for 22.5 wt % 6FDA+ 7.5 wt% ODPA-PDODA-PEG1500 polyimide after imidization process (42.1 wt % of PEG1500)

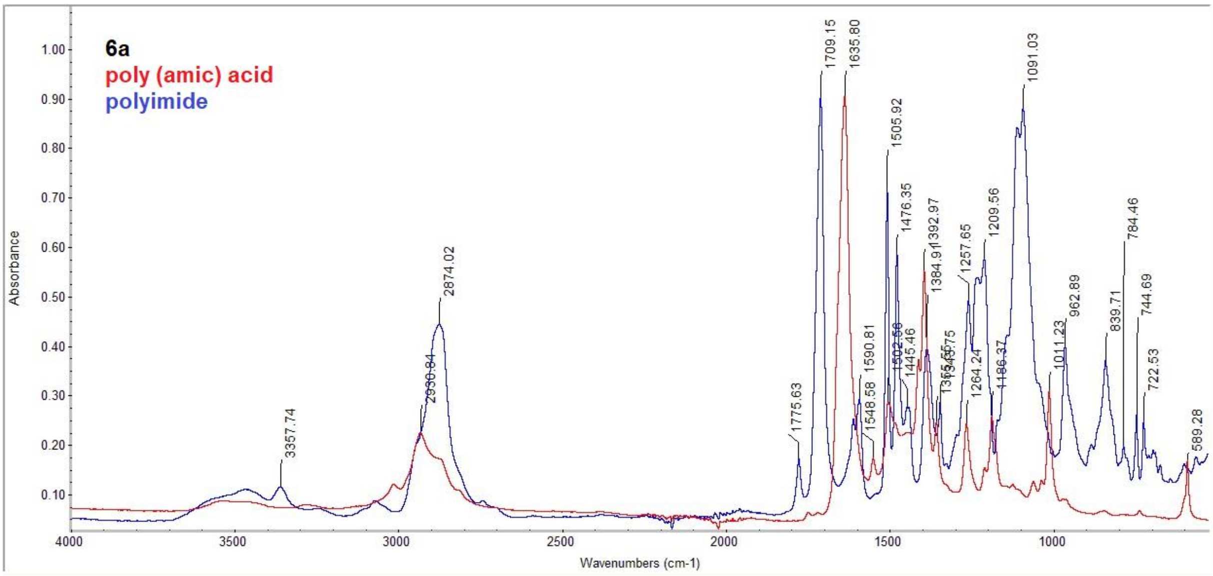

Figure S6a. Comparison of FTIR of 15 wt % 6FDA+ 15 wt% ODPA-PDODA-PEG1500 poly (amic acid) precursor and polyimide after imidization process (42.1 wt % of PEG1500) with given pick values.

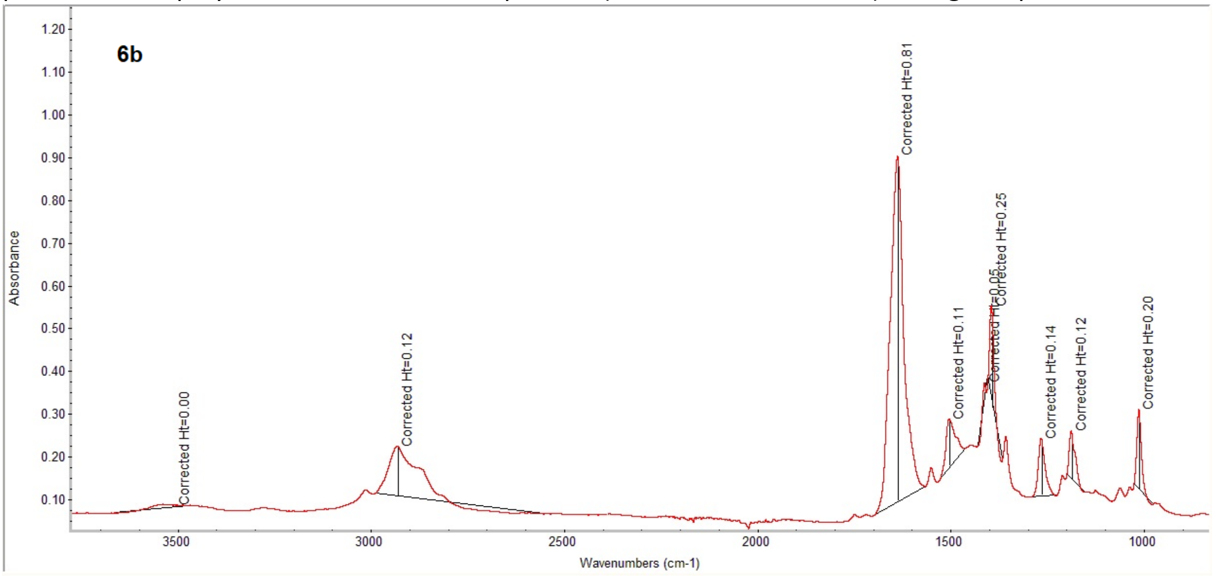

Figure S6b. Height of the picks for 15 wt % 6FDA+ 15 wt% ODPA-PDODA-PEG1500 poly (amic acid) (42.1 wt % of PEG1500) precursor

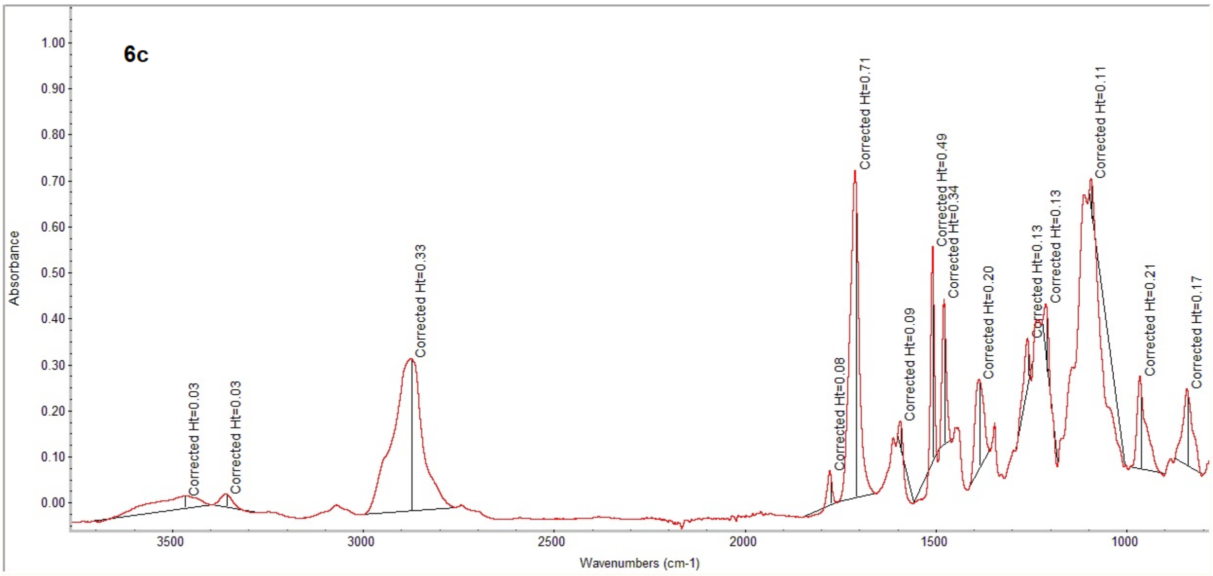

Figure S6c. Height of the picks for 15 wt % 6FDA+ 15 wt% ODPA-PDODA-PEG1500 polyimide after imidization process (42.1 wt % of PEG1500)

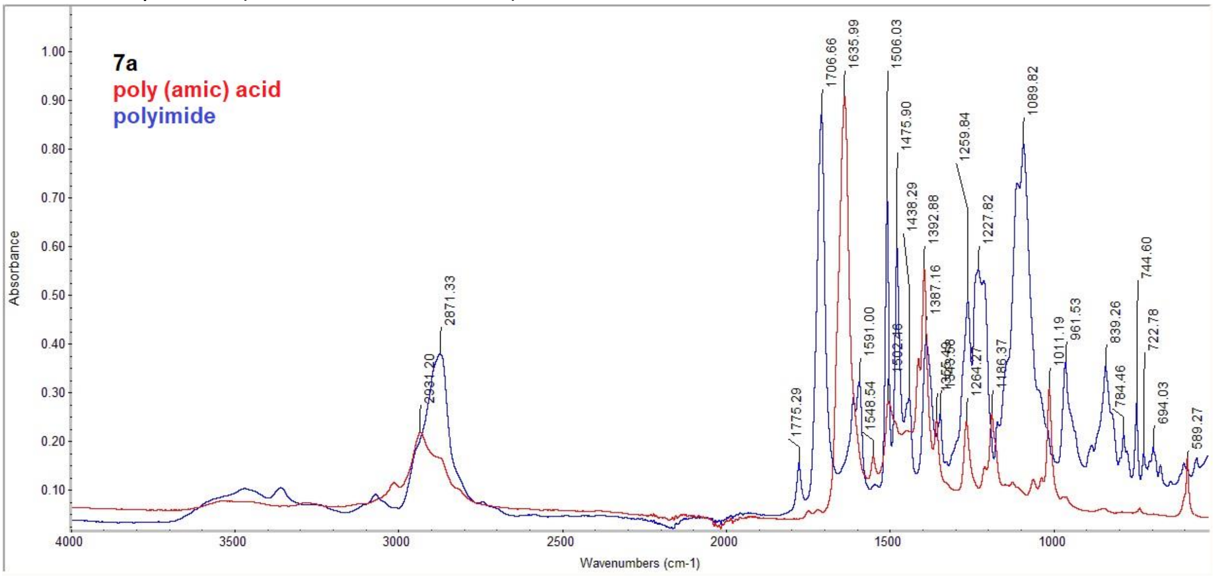

Figure S7a. Comparison of FTIR of 7.5 wt % 6FDA+ 22.5 wt% ODPA-PDODA-PEG1500 poly (amic acid) precursor and polyimide after imidization process (42.1 wt % of PEG1500) with given pick values.

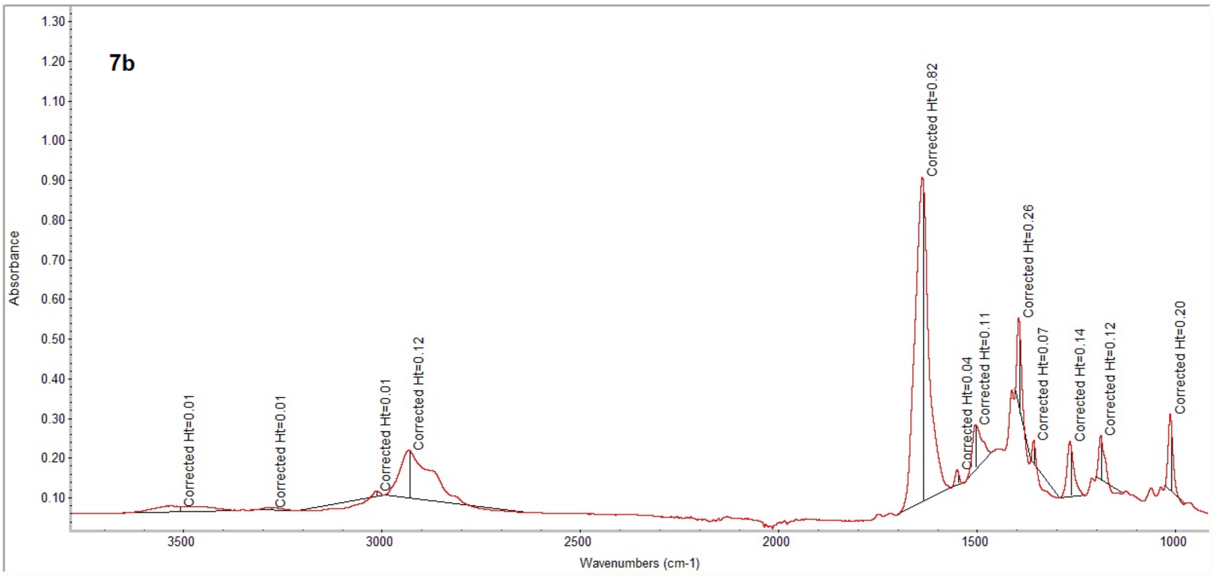

Figure S7b. Height of the picks for 7.5 wt % 6FDA+ 22.5 wt% ODPA-PDODA-PEG1500 poly (amic acid) (42.1 wt % of PEG1500) precursor

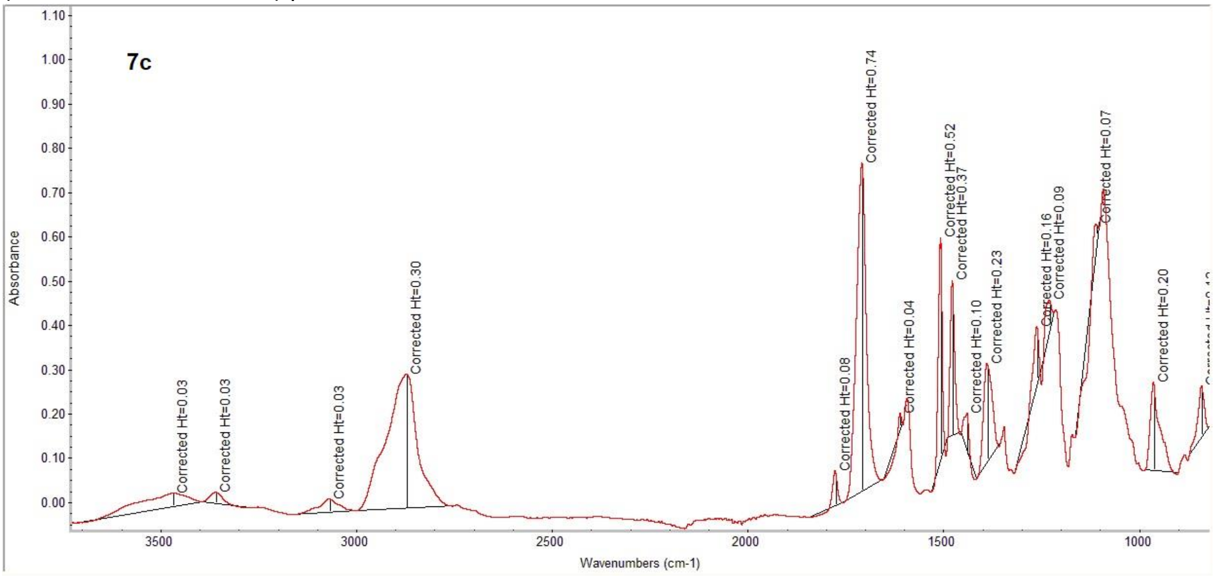

Figure S7c. Height of the picks for 7.5 wt % 6FDA+ 22.5 wt% ODPA-PDODA-PEG1500 polyimide after imidization process (42.1 wt % of PEG1500)

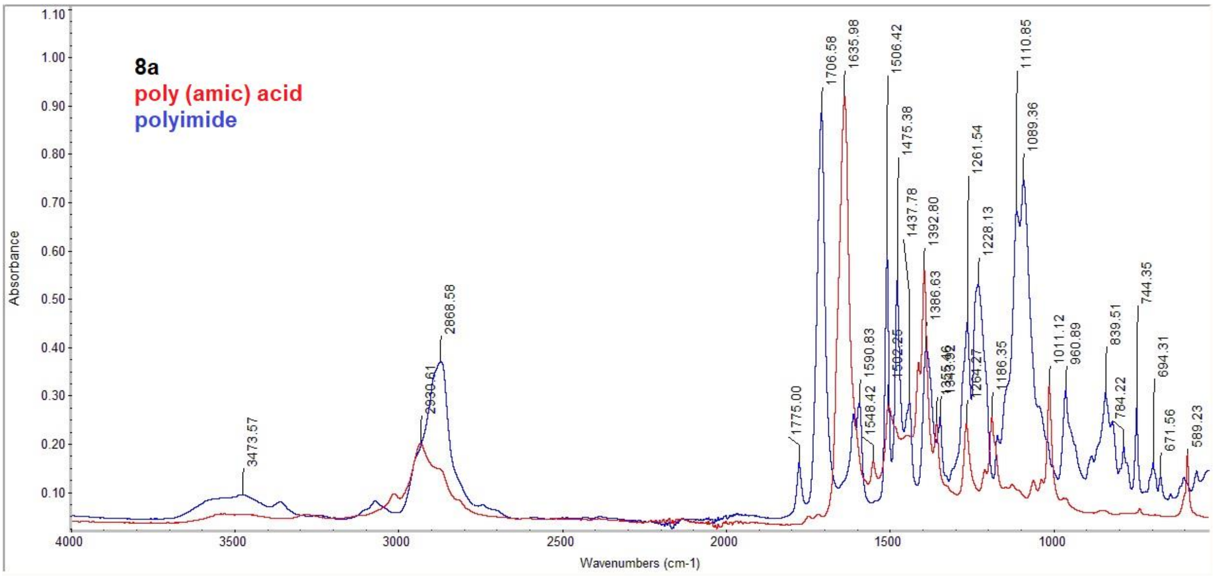

Figure S8a. Comparison of FTIR of 0 wt % 6FDA+ 30 wt% ODPA-PDODA-PEG1500 poly (amic acid) precursor and polyimide after imidization process (42.1 wt % of PEG1500) with given pick values.

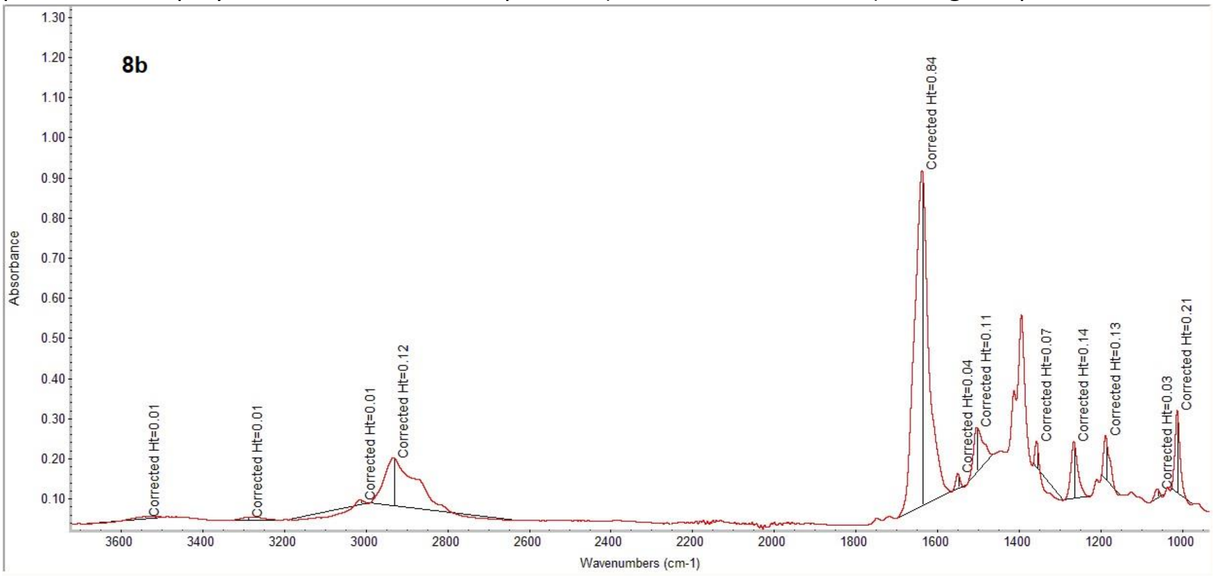

Figure S8b. Height of the picks for 0 wt % 6FDA+ 30 wt% ODPA-PDODA-PEG1500 poly (amic acid) (42.1 wt % of PEG1500) precursor

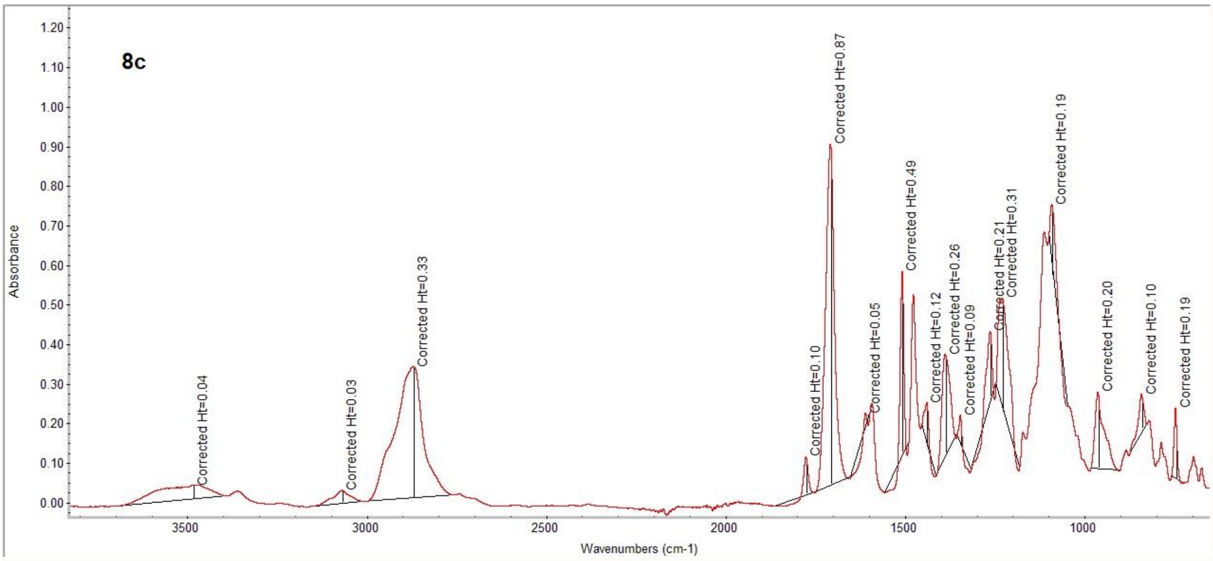

Figure S8c. Height of the picks for 0 wt % 6FDA+ 30 wt% ODPA-PDODA-PEG1500 polyimide after imidization process (42.1 wt % of PEG1500)

Table S1. Detailed calculations of conductivity of the synthesized SBC membrane

**6FDA-ODPA-PEG1500 (42.1wt%) 60°C & RH70%**					
thickness (cm)	0.02	0.05	0.03	0.02	0.016
width (cm)	0.7	0.5	0.5	0.7	0.3
resistivity (ohm)	302.9	172	453.8	353.1	1273.33333
conductivity (mScm^{-1})	100.221667	98.8372093	62.4357279	85.9732168	69.5353403
average	83.4006322				
standard variation	17.026647				
